# A Reversible and Dynamic Surface Functionalization for Fluidity Controlled Multivalent Recognition of Lectins and Bacteria

**DOI:** 10.1002/advs.202416658

**Published:** 2025-04-26

**Authors:** Thomas Hix‐Janssens, Adam Tillo, Hanna Isaieva, Zita Lopes da Silva, Zahra Fatahi, Michele Larocca, Gustav Sedelius, Sara Björk Sigurdardóttir, Yulia Sergeeva, Tiba Al‐Dujaili, Julia R. Davies, Kushagr Punyani, Börje Sellergren

**Affiliations:** ^1^ Biofilms Research Center for Biointerfaces Department of Biomedical Science Faculty of Health and Society Malmö University Malmö 205 06 Sweden; ^2^ Section for Oral Biology and Pathology Faculty of Odontology Malmö University Malmö 205 06 Sweden; ^3^ Surecapture Technologies AB Forskaren 1, Per Albin Hanssons väg 35 Malmö 21432 Sweden; ^4^ Spermosens AB The Spark Medicon Village Lund Sweden

**Keywords:** bacterial recognition, membrane mimic, multivalent receptor, rewritable surfaces, rSAM

## Abstract

The paper reports the design of multivalent bacterial receptors based on reversible self‐assembled monolayers (rSAMs) on gold and glass substrates, mimicking the ligand display on host cells and extracellular matrices. The layers consist of α‐(4‐amidinophenoxy)alkanes decorated at the ω‐position with β‐galactose (Gal) or sialic acid (SA). The former acts as a mobile ligand binding to the complementary adhesin, LecA, a key virulence factor of the multi‐drug‐resistant bacterium *Pseudomonas aeruginosa* (PA). Binary amphiphile mixtures containing either of these ligands, spontaneously self‐assemble on carboxylic acid terminated SAMs on gold or glass surfaces to form rSAMs that are easily tunable with respect to the ligand ratio. It is shown that this results in the ability to construct multi‐reusable surfaces featuring strong affinity for the bacterial adhesin and recognitive surfaces for bacteria, the latter demonstrated by incubating a culture of PA or the oral commensal species *Streptococcus gordonii* (SG) on either Gal or SA functionalized rSAMs. In contrast to the mobile ligand display, surfaces featuring covalently attached “static” ligands exhibited low LecA affinity. This approach to wet chemical surface functionalization is unique in imparting both rapid restorability and adaptability, the latter compatible with heteromultivalent receptor designs for boosting lectin and bacteria affinity and specificity.

## Graphical abstract

A reversible wet chemical surface functionalization results in dynamic multivalent receptors showing ligand‐specific high affinity for lectins and bacteria.

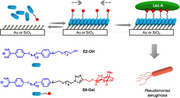

## 1 Introduction

The adhesion of microorganisms to surfaces is the focus of a broad range of efforts aimed at understanding, detecting, and preventing infections.^[^ ^1^, ^2^ ^]^ This ranges from studies of surface attachment of bacteria or viruses to host cells or abiotic surfaces, and its consequences for virulence and the mechanisms of biofilm formation,^[^ ^3^, ^4^, ^5^, ^6^, ^7^ ^]^ to the design of recognitive surfaces for use in sensors and in clinical diagnostics.^[^ ^8^, ^9^, ^10^, ^11^, ^12^, ^13^ ^]^ Surface dynamic features in terms of stiffness and membrane fluidity have been suggested to play a role in this context.^[^ ^3^, ^14^, ^15^ ^]^ The mode of adhesion is different when comparing hard abiotic surfaces with soft surfaces of host cells. While bacterial attachment to abiotic surfaces is governed by general physicochemical surface characteristics (e.g., wettability, charge, topography/roughness, hydrophobicity), effectively studied using defined self‐assembled monolayers (SAMs),^[^ ^16^ ^]^ surface attachment to host cell membranes involves specific multivalent interactions typically between multiple bacterial adhesins and specific glycans on the host cell glycocalyx.^[^ ^1^ ^]^ In both cases, the adhered bacteria can chemically and mechanically sense their microenvironment, impacting their behavior in terms of virulence and biofilm formation. Indeed, the stiffness of an abiotic surface and host cells as well as membrane fluidity have been suggested to influence adhesion and virulence.^[^ ^13^, ^14^ ^]^

Beyond the above fundamental aspects, access to techniques allowing rapid and reversible attachment of bacteria selective coatings can meet urgent needs in clinical diagnostics for sensing or reversible bacteria capture. Here, biomimetic sensors based on multivalent receptors employing glycans as recognition elements constitute an interesting antibody‐free alternative for detection of bacteria.^[^ ^17^, ^18^, ^19^, ^20^, ^21^, ^22^ ^]^ A distinguishing feature of these receptors is their ability to effectively exploit binding amplification due to multivalency. For glycans conjugated to rigid scaffolds, such as conformationally defined peptides, the distance between ligands has to be carefully tuned^[^ ^23^ ^]^ or valency increased to high numbers through multistep synthetic protocols^[^ ^24^ ^]^ for this amplification to occur. Alternatively, the glycans can be introduced as tri‐ or higher order valent head groups in SAMs.^[^ ^21^ ^]^ Despite impressive amplifications in terms of lectin affinity, one limitation of the above strategies is the reliance on covalent ligand attachment and hence absence of membrane‐like ligand mobilities.

**Figure 1:**
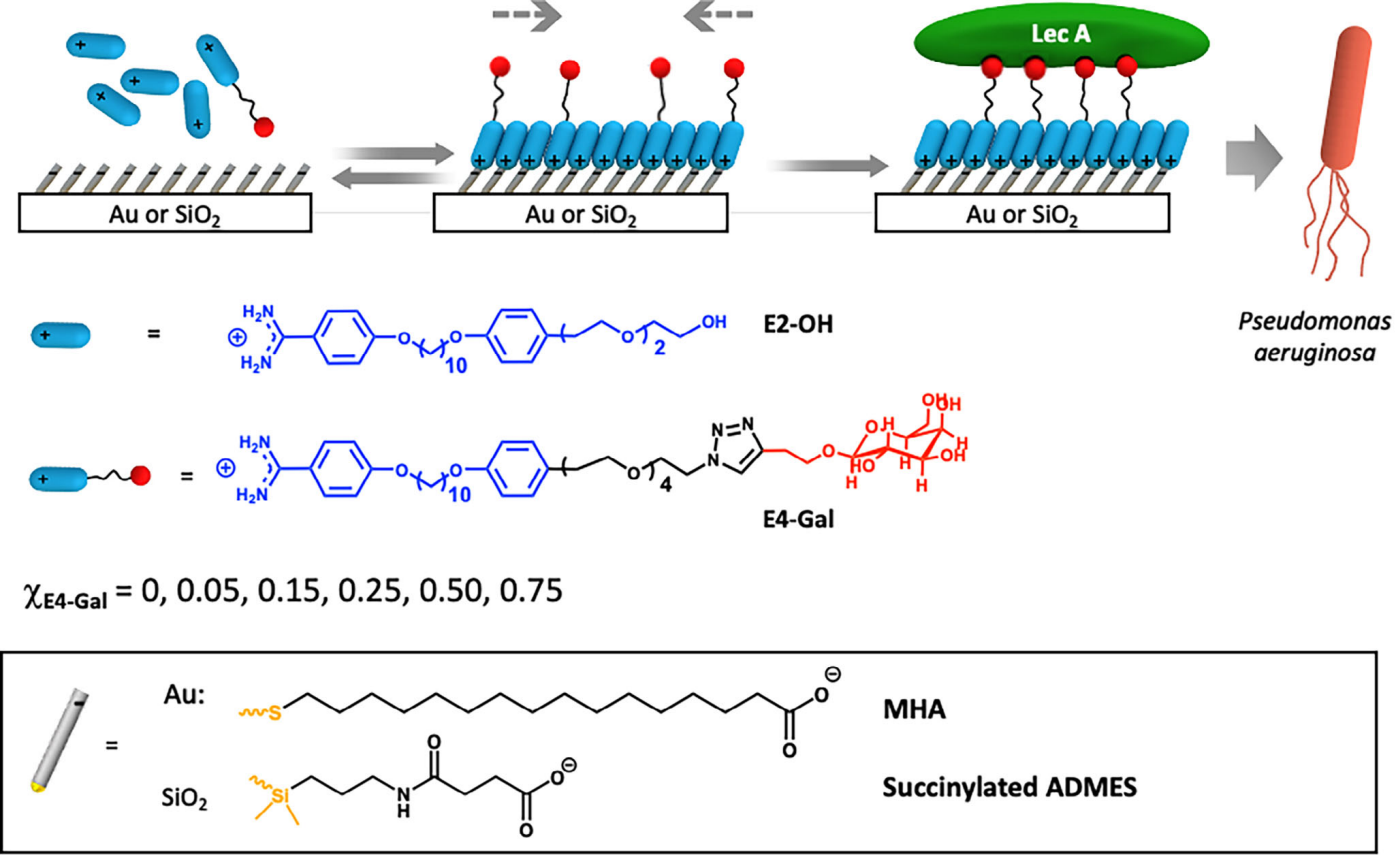
Scheme showing the composition and preparation of rSAM‐based fluidic surfaces on gold or glass for protein and bacterial recognition. The pH‐switchable mixed rSAMs are formed by self‐assembly of the indicated amidines at slightly basic pH on anchor SAMs of mercaptohexadecanoic acid (MHA) on gold or succinylated‐ADMES (3‐aminopropyldimethylethoxysilane) modified glass, and then used for LecA and PA recognition.

The forementioned needs provide incentives for developing versatile cell membrane mimetics allowing control of both ligand presentation and membrane fluidity. We believe that our membrane mimetic reversible self‐assembled monolayers (rSAMs) offer a way forward (**Figure** **1**).^[^ ^25^, ^26^, ^27^, ^28^, ^29^ ^]^ These are prepared by the spontaneous pH‐switchable self‐assembly of mixtures of benzamidine‐based bola amphiphiles on top of carboxylic acid terminated SAMs from neutral or slightly basic aqueous solutions. The amphiphile mixture comprises an inert filler amphiphile and one or more ligand‐decorated amphiphiles allowing mixing ratio‐based fine tuning of target binding affinity and selectivity for capture, inhibition, or sensing. Fluorescence recovery after photobleaching (FRAP) measurements on fluorescently doped rSAMs revealed diffusivities in the range of supported lipid bilayers (ca. 1 µm^2^ s^−1^) and binding affinities strongly depending on amphiphile diffusivity.^[^ ^26^ ^]^ Hence, mobile ligands can lead to an enhancement of affinity exceeding 4 orders of magnitude. Combined with nanoplasmonic sensor platforms this can boost sensitivity to very high levels.^[^ ^28^ ^]^

rSAMs can also be used as a versatile tool to study and control cell adhesion and differentiation.^[^ ^27^ ^]^ Incorporation of the tripeptide RGD as cell adhesive ligands in two rSAMs featuring different anchors, and hence stiffness, allowed cell shape and morphology to be influenced. Dynamic control over surface composition, achieved by addition of inert filler amphiphiles to the RGD‐functionalized rSAMs, reversed the cell adhesion process. These results together demonstrate that rSAMs are unique in being able to both modulate cell adhesive behavior and capture and subsequently release cells in a non‐invasive manner. In order to explore these features in terms of bacterial adhesion we have here focused on *Pseudomonas aeruginosa* (PA), a Gram‐negative aerobic rod‐shaped bacterium that typically infects patients with underlying medical conditions or compromised immune systems.^[^ ^30^ ^]^ The infections pose a serious health threat due to multidrug resistance and cause symptoms such as pneumonia, urinary tract infections, septicemia, and surgical site infections. PA carries two surface adhesins, LecA and LecB, that play a key role in the adhesion of the bacterium and are determinants of PA virulence.^[^ ^31^ ^]^ LecA has the highest affinity for D‐galactose, while LecB, a generally less specific lectin, shows strong binding to L‐fucose and D‐mannose. Despite having different amino acid sequences, both PA lectins show a similar quaternary structure, being homotetramers with four carbohydrate binding sites. In LecA, the distance between cavities is 32 and 70 Å, while the more globular LecB binds sugars in the distance of 43 and 45 Å.

This report shows that rSAMs can be used as a pH‐switchable surface modification for recognition of LecA and PA and for multiple reuses of a single surface. It is demonstrated that the surfaces can be conveniently engineered to display high‐affinity binding of adhesins exceeding that of their covalently‐linked counterparts. The results suggest a highly versatile approach to engineer surfaces for discrimination and sensing of pathogens and for fundamental studies of bacterial adhesion.

## 2 Results and Discussion

### 2.1 rSAM design for Bacterial Lectin Recognition

**Figure 2:**
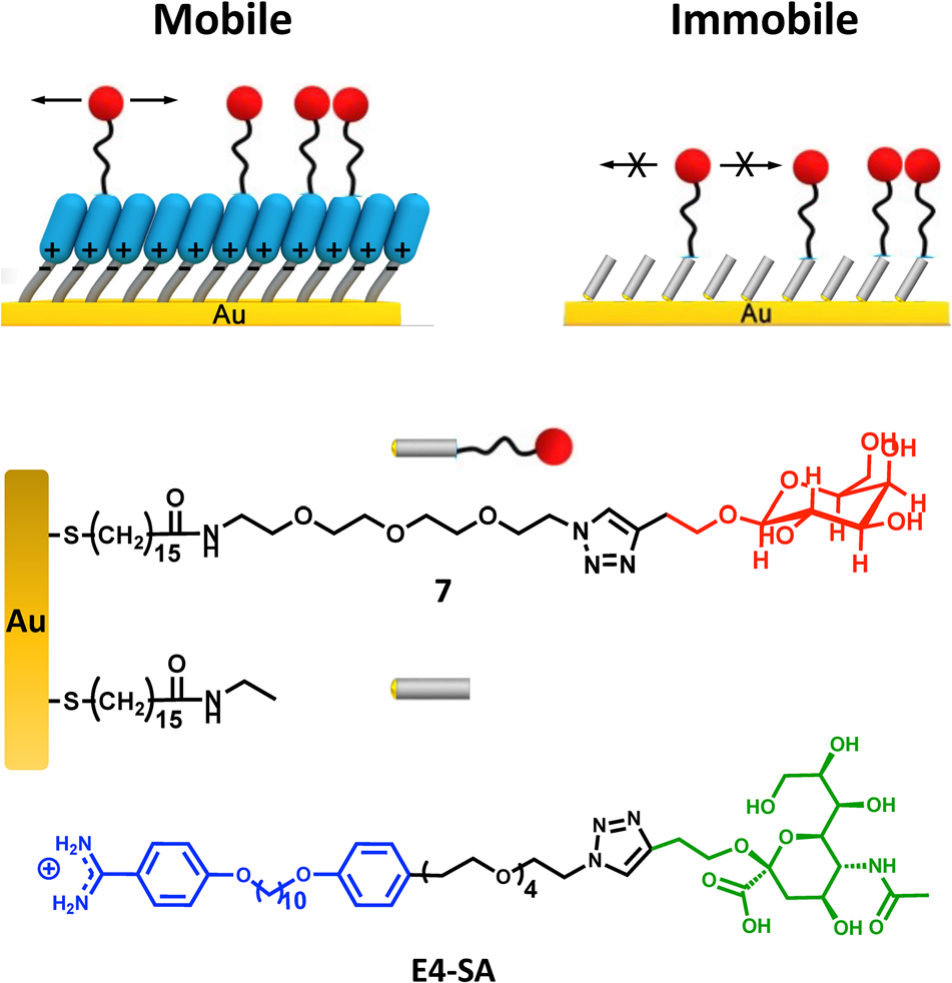
Design of controls for studying the effect of ligand mobility (7) or ligand identity (E4‐Gal versus E4‐SA). The Gal functionalized SAM was prepared by EDC/NHS catalyzed coupling of 7 to a SAM of MHA on gold (SAM‐Au) and used as an immobile control for assessing the role of ligand mobility in relation to the corresponding rSAM. The arrows indicate the presence of mobile or immobile (crossed arrows) ligands.

Optimization of ligand‐decorated SAMs demands attention to multiple factors governing the multivalent interactions with the receptor. Key parameters are the nature of the ligand, the length of the tether connecting the ligand head group and mesogen unit, and the surface density of ligand amphiphiles. Previously, it was demonstrated that optimal hemagglutinin binding to sialic acid (SA) rSAMs was achieved using a tether containing four ethylene glycol repeating units and a ligand density of 15%.^[^ ^25^ ^]^ Using these specifications as a starting point β‐galactose terminated amphiphile E4‐Gal was synthesized by a Huisgen‐type click‐coupling between an alkyne derivative of β‐galactose and an azide‐terminated amidine following our previously reported procedure^[^ ^32^ ^]^ (see Supporting Information). The alkyne derivative of β‐galactose was obtained following a two‐step procedure from commercially available β‐D‐galactose pentaacetate (Scheme S1, Supporting Information). β‐D‐Galactose 3‐butyn‐1‐ol was subsequently coupled to benzamidine **4** or amine **6** (Scheme S2, Supporting Information) via the copper‐catalyzed azide‐alkyne cycloaddition. The galactose‐terminated E4‐Gal **5** and amine‐Gal **7** were isolated as pure β‐anomers, as confirmed by ^1^H and ^13^C NMR analysis (Figures S11–S24, Supporting Information) and measurements of their optical rotation. E4‐Gal combined with E2‐OH constituted the binary amphiphile system used to tune LecA and PA affinity and selectivity (Figure 1). Two control surfaces were also introduced, the first by replacing E4‐Gal with E4‐SA and the second featuring covalently linked β‐Galactose to probe the effect of ligand mobility (**Figure** **2**).

### 2.2 β‐Gal and Filler Amidines Form Dynamic LecA Receptors on MHA‐SAMs on Gold

**Figure 3:**
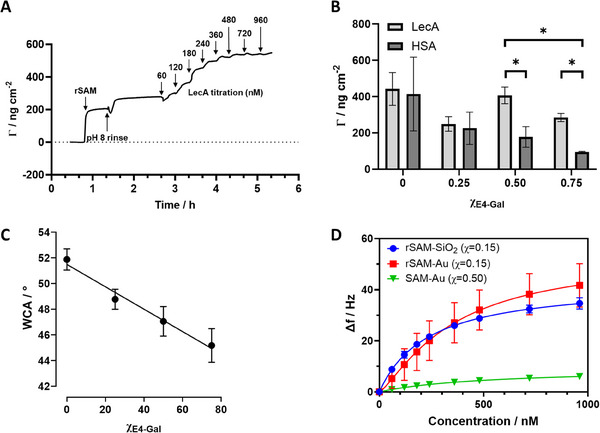
A) Titration of the galactose‐presenting rSAM (χ**_E4‐Gal_** = 0.50) with increasing concentrations of LecA monitored by QCM‐D. The adsorbed mass was modeled by the Sauerbrey equation. The injection steps are indicated by arrows. B) Limiting adsorbed mass after addition of LecA or HSA on mixed rSAMs with different E4‐Gal contents. (* = p < 0.05) C) Water contact angles versus theoretical rSAM composition. Mean and standard deviation calculated from at least three measurements. D) Frequency change upon titration of the galactose presenting rSAMs (χ**_E4‐Gal_** = 0.15) on modified quartz or gold chips versus a SAM with covalently attached Gal (7) (SAM‐Au). The data were fitted with the Langmuir 1:1 or Hill binding models.

The study first focused on a proven protocol based on the use of mercaptohexadecanoic acid (MHA) SAM modified gold surfaces as negatively charged rSAM anchors. This was followed by the formation of a series of mixed rSAMs from solutions containing different mole fractions of E4‐Gal and filler E2‐OH. These surfaces were evaluated by quartz crystal microbalance (QCM) (**Figure** **3**), infrared reflection‐adsorption spectroscopy (IRRAS), and goniometry. Table S1 (Supporting Information) and Figure 3C show the water contact angles (WCA) measured after each step of the gold surface modification. The contact angle of the MHA SAM is consistent with our previous results and indicates formation of a moderately hydrophilic surface. The rSAMs exhibited a more hydrophobic nature with the highest WCA observed for single component rSAM of E2‐OH and a linear decreasing trend when increasing the ratio of E4‐Gal, all in line with our previous observations. The linear dependence of the contact angle on the composition of the amphiphile solution (Figure 3C) agrees well with previous studies of silane‐ or thiol‐ based SAMs indicating formation of mixed monolayers with stoichiometrically incorporated amphiphiles.^[^ ^33^ ^]^ Further support for the latter will be discussed below.

IRRAS was then used to further confirm the presence, order, and orientation of both rSAM amphiphiles adhered to the thiol SAM. Figures S1 and S2 (Supporting Information) show the significant peaks of neat E4‐Gal, the anchor SAM and rSAMs formed from the two amphiphiles. The formation of well‐ordered MHA and amidine monolayers is indicated by CH_2_ vibrational mode peaks at 2918 cm^−1^ (asymmetric C─H bond stretching) and 2850 cm^−1^ (symmetric stretch) (Figure S2, Supporting Information). The overall higher peak intensities observed in the rSAM spectra compared to that of the anchor SAM supports the presence of a second dense monolayer film. The sharp absorption band assigned to the carboxylate head group at 1715 cm^−1^ was replaced by a more complex set of peaks in the low frequency region upon rSAM modification. The benzamidines were distinguished by observing the strong NH‐ and OH‐stretch bands at 3100–3200 and 3300–3500 cm^−1^, the C‐H stretch bands of the alkyl chains at 2929, 2918, and 2850 cm^−1^, the sharp and intense aromatic (C═C)_1,4_ stretching mode signals of the bola amphiphiles at 1612, 1513, and 1491 cm^−1^, and the C‐O‐C stretch bands at ca. 1250 and 1000–1150 cm^−1^.^[^ ^34^ ^]^ Comparing the spectra of the mixed rSAMs we noted that the band integrals of the OH‐stretch band at 3300–3500 cm^−1^ and the ether C─O stretch vibration at 1000–1150 cm^−1^ increased with an increasing χ**_E4‐Gal_**. This agrees with the amphiphile's longer tether, containing four ethylene glycol repeats, and its OH rich headgroup. A nearly linear increase in the intensities and peak integrals with increasing χ_E4‐Gal_ were observed for the 3300–3500 cm^−1^ band (Figure S3, Supporting Information). This adds experimental support for an unbiased incorporation of E4‐Gal into the rSAM reflecting the solution stoichiometry.

Formation of the rSAM layer and its interaction with model proteins were then followed in situ using the QCM‐D technique. Recorded changes in frequency (*f*) and dissipation (*D*) were used for calculation of changes in adsorbed mass. Gold‐coated quartz sensors were modified ex situ with MHA, mounted in the QCM‐D instrument and equilibrated with HEPES buffer, until stable *f* and *D* values were obtained. Injection of an amidine solution with χ_E4‐Gal_ = 0.50 led to an immediate drop in *f* with a concurrent small increase in *D*, corresponding to formation of a second amphiphile layer on top of the MHA SAM (Figure 3A; Figure S6, Supporting Information) with an adsorbed Sauerbrey mass of 300‐360 ng cm^−^^2^, which is in accordance with our previous report^[^ ^26^ ^]^ and reflects formation of a monolayer of densely packed amphiphiles. The surfaces were further equilibrated by washing with additional amounts of HEPES buffer.

After obtaining stable *f* and *D* values, LecA solutions of increasing concentrations were injected which were accompanied by a decrease in *f* and an increase in *D* indicating attachment of the proteins and an increase in the adsorbed Sauerbrey mass levelling off at ca. 540 ng cm^−^^2^. This is consistent with formation of a dense protein film slightly exceeding a monolayer.^[^ ^35^ ^]^ After surface saturation the sensors were regenerated by washing with an excess of pH = 2.0 buffer leading to an abrupt return of *f* and *D* to approach the starting values.

Commonly, change in mass or thickness can be estimated with simplified Sauerbrey equation only for experiments where changes in dissipation are lower than 5% of frequency shifts. Higher dissipation shifts or extensive frequency spread for different overtones require use of viscoelastic modeling. Comparing Sauerbrey and Voigt modeling resulted in similar values for the adsorbed mass with the former consistently exceeding the latter by ca. 10%. For instance, the adsorbed mass at 960 nm LecA was estimated to 540 ng cm^−^^2^ using Sauerbrey and 480 ng cm^−^^2^ using Voigt modelling. This confirms a densely packed arrangement of the rSAM layer and only a small contribution from the coupled water inside of the film. Underestimation of the mass (or thickness) by the Sauerbrey equation occurs for surfaces where the viscous component of the film dominates over the elastic component, whereas slightly higher values of the Sauerbrey mass may in turn suggest domination of the elastic component.^[^ ^36^ ^]^

Different compositions of the rSAMs were then compared in terms of their ability to bind LecA and resist non‐specific adsorption using human serum albumin (HSA) as a reference protein. Both proteins adsorbed non‐specifically to the rSAM of the E2 filler alone. With only two ethylene glycol repeats, the rSAM of this filler alone is more hydrophobic (Figure 3C) and poorly protein resistant. Increasing the molar fraction of the E4‐Gal (χ_E4‐Gal_ = 0.25; 0.50; 0.75) then resulted in the adsorbed mass of LecA peaking at χ_E4‐Gal_ = 0.50. In parallel, the HSA adsorption displayed a decreasing trend with increasing E4‐Gal content (Figure 3B). This agrees with HSA's affinity for hydrophobic surfaces^[^ ^37^ ^]^ and parallels the decreasing trend of WCAs measured for these surfaces (vide infra). Interestingly, the adsorption mode, i.e., hydrophobically driven adsorption versus ligand‐receptor driven, reflects on the adsorption kinetics. Whereas adsorption of both proteins to the E2‐rSAM (and HSA to all surfaces), is slow with no apparent saturation reached within the time interval of the measurement, adsorption of LecA to the E4‐Gal surfaces occurs with much faster on rates resulting in saturation within the measured time interval.

Attempts to prepare rSAMs from solutions of pure E4‐Gal did not yield stable rSAMs (results not shown). In this case, the initial steep increase in thickness after injection of amphiphile was followed by quick loss of adsorbed amidines in the conditioning step, resulting in a very low final thickness. These observations are in line with our previous reports and can be ascribed to the bulkiness of the sugar head groups which hinders formation of well‐ordered monolayers.^[^ ^26^ ^]^

The composition resulting in the maximum LecA combined with minimum HSA absorption (χ_E4‐Gal_ = 0.50) was investigated further. A measurable response in terms of *f* and *D* was observed for concentrations as low as 60 nm of lectin. Further titration resulted in a continued decrease in frequency which finally levelled off at a concentration of 500–600 nm. Fitting the slightly sigmoidal binding curve with the Hill‐Langmuir binding isotherm model resulted in an affinity constant (K_d_) of 319 ± 5 nm. Furthermore, the QCM‐D titration of this surface showed very close values for each frequency overtone, indicating a well‐ordered and thin monolayer (Figure S6, Supporting Information). Hence, the rSAM with χ_E4‐Gal_ = 0.50 was chosen for further experiments. The designed bilayer surface showed a strong signal response to LecA and notably reduced non‐specific binding of HSA (Figure 3B). Thus, titration of the rSAM with HSA resulted only in weak frequency shifts for initial aliquots of the analyte (60 nm) followed by a lack of response to higher concentrations (Figure S7, Supporting Information).

The above was supported by IRRAS (Figure S4, Supporting Information) showing a pronounced increase in the amide I (1620–1710 cm^−1^) and NH/OH stretch (3100‐3500 cm^−1^) band intensities (Figure S4A,B, Supporting Information) for LecA in contact with the Gal modified surfaces whereas the rSAM of the E2‐OH filler alone gave nearly identical band intensities before and after LecA incubation indicating only minor nonspecific binding (Figure S4C, Supporting Information). This disagrees with the QCM‐D data in Figure 3B showing the opposite behaviour but agrees with the plate binding assay results (vide infra). The former discrepancy can be attributed to the higher protein concentration (960 nm) and longer incubation times (2.5 h) used in the QCM‐D experiments. The LecA specificity was clearly demonstrated by the significantly lower intensities for HSA and LecB, in the latter case indicating a near complete rejection of the protein. To investigate whether the ligand‐mediated binding influenced protein conformation or orientation with respect to free protein, deconvolution by Gaussian curve fitting was attempted for estimating the relative contribution of the different secondary structures (Figure S4D, Supporting Information).^[^ ^38^ ^]^ Table S3 (Supporting Information) provides an estimate of the relative contributions of the different motifs for surface bound protein. In agreement with the LecA crystal structure^[^ ^39^ ^]^ the fitting indicated predominance of β‐sheets, random coils and β‐turns with minor contribution from α‐helices. The distribution of the structural motifs roughly agreed with the crystal structure and no influence of the ligand density on the distribution was noted.

The high LecA affinity displayed by the rSAM with χ**_E4‐Gal_** = 0.50 contrasts with the weak binding observed using the sensor chip with covalently attached ligand **7** (Figure 3D). In this experiment, β‐Gal terminated amine **7** and ethanolamine as a filler were covalently coupled to the surface as shown in Figure 2. The Gal‐appended SAM displayed a significantly lower response to the analyzed lectins compared to the corresponding rSAM. The latter showed a steep response to LecA, a high binding affinity (vide supra) and a low limit of detection. In contrast, QCM‐D analysis of the covalently modified surface resulted in a very weak signal response and a significantly lower binding affinity.

The above results agree with recent literature reports. Studies of other Gal‐presenting SAMs have shown similar affinity constants as observed for the SAM‐appended Gal in this work.^[^ ^40^, ^41^ ^]^ On the other hand, the rSAM surface displayed a K_d_ close to the value reported for a polyproline scaffold carrying Gal ligands precisely spaced at 26 Å distance, to match the binding site separation in the tetrameric protein.^[^ ^23^ ^]^ All in all and in line with our previous observations,^[^ ^26^ ^]^ this strongly suggests that dynamic interactions in rSAMs play an important role in enhancing LecA‐surface interactions. This allows the β‐Gal ligands to adapt to the multivalent target and engage in fully developed selective multivalent interactions.

### 2.3 rSAMs Anchored on Glass and Gold Show Similar Surface Coverage, Order, and LecA Affinity

**Figure 4:**
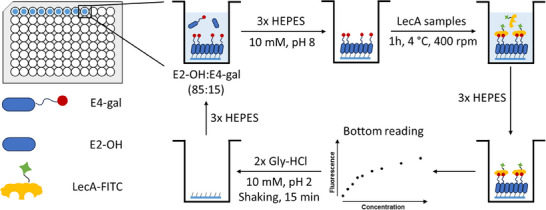
Principle of rSAM‐ and fluorescence‐based protein binding assay including multi‐reusable glass disks.

Glass substrates, like microscope slides and cover slides, are widely used in biology and are often combined with various surface modifications to adjust their interactions with biological materials.^[^ ^42^ ^]^ As for gold, SAMs on glass are ideal for fine‐tuning such interactions. Formation of SAMs on glass often requires multiple processing steps ending with irreversible silanization reactions using alkoxy‐ or chlorosilanes in anhydrous organic solvents. The subsequent covalent chemistry used to further functionalize the surface often prevents its reuse and leads to lengthy procedures and a waste of substrates and reagents. A reversible surface modification with rSAMs would considerably facilitate the handling in this regard. Moreover, it allows the rSAM‐based receptors to be prepared on optically transparent surfaces compatible with microplate or microarray based parallel assays of both protein binding and bacterial adhesion.^[^ ^23^ ^]^ This was anticipated to significantly facilitate system optimization. To achieve this, silane‐based anchor SAMs were prepared on circular glass disks with diameters precisely fitting the wells of 96‐ or 6‐well microplates (**Figure** **4**), the former for tests of FITC‐labelled protein binding and the latter for testing the affinity of the surfaces for planktonic bacteria.

The substrate handling procedure involving both the ex situ and in situ steps is schematically shown in Figure 4. All disks were first modified with a carboxylic acid anchor SAM by aminosilanization with the monoalkoxysilane ADMES followed by reaction of the surface amines with succinic anhydride.^[^ ^43^ ^]^ This two‐step procedure offers a robust and simple route to ordered carboxylic acid functionalized SAMs. The SAM‐modified disks were thereafter immersed in the amidine solution followed by characterization of their structural and surface properties by FTIR (Figure S5, Supporting Information), as well as tests of their protein affinity using a multifunctional microplate reader.

**Figure 5:**
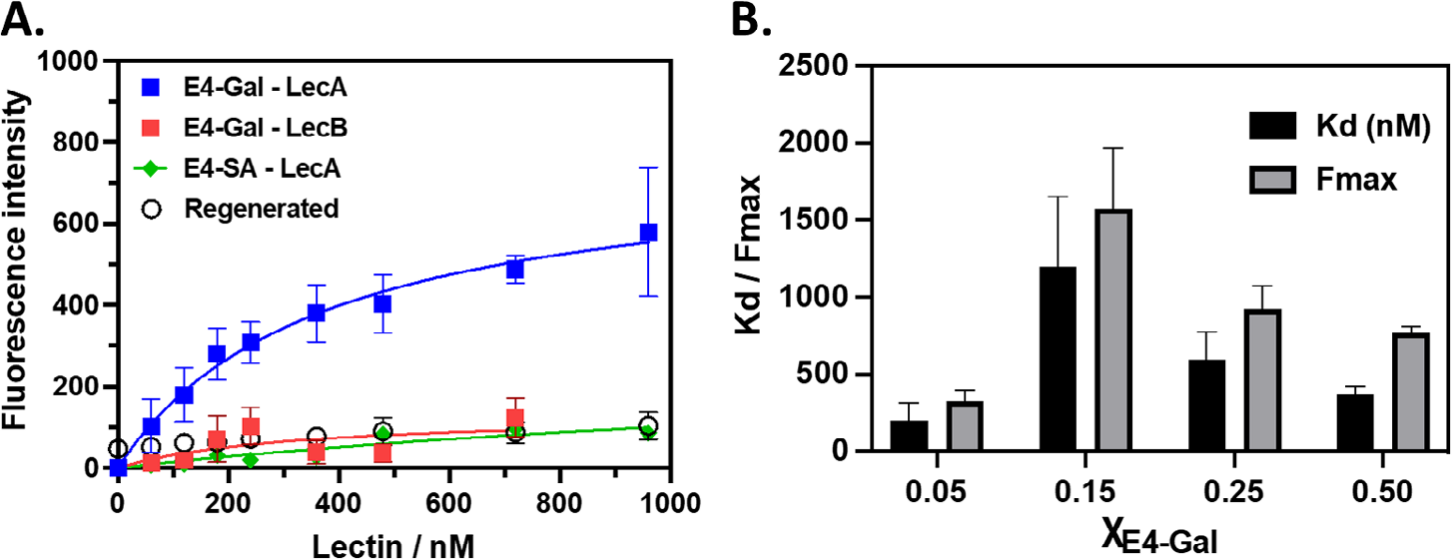
Lectin recognition using reusable glass disks. A) Bottom‐mode fluorescence of rSAM modified glass cover slips after overnight incubation with amidine solutions containing χ_E4‐Gal_ = 0.50, followed by incubation with LecA (blue and green curves) or LecB (red curve). Residual fluorescence was also checked after rinsing with acidic buffer (open circles). λ_ex_ = 495 nm, slit width 5 nm; λ_em_ = 519 nm, slit width 7.5 nm. Results show average with standard deviation from three independent repeats (*n* = 3) on the same cover slip. B) Fitting parameters (equilibrium dissociation constant K_d_; fluorescence maximum F_max_) from curve fitting using a Langmuir mono‐site binding model of the data in (A) and Figure S9 (Supporting Information) (see Table S4, Supporting Information).

The interactions of these rSAMs with FITC‐labeled model proteins were thereafter tested and compared with a QCM‐D test of a corresponding rSAM anchored on quartz (Figure 3D). First, the surfaces were titrated with LecA followed by rinsing of the disks and measurement of residual fluorescence using bottom‐mode reading. This was followed by regeneration of the surfaces by a brief rinsing step with Gly‐HCl pH 2 buffer to remove the rSAM. Thereafter, re‐equilibration in the binding pH 8 buffer, rSAM formation was renewed, and the titration procedure repeated (Figure 4). In this way, the same glass disks were used in three consecutive binding experiments. The average intensities with SDs are plotted in **Figures** **5** and S9 (Supporting Information) with binding parameters from nonlinear regression assuming a Langmuir mono‐site model listed in Table S4 (Supporting Information).

It was gratifying to observe that this parallel readout technique resulted in affinities for the model proteins (Figure 5, Figure S9 and Table S4, Supporting Information) and their relative surface binding preferences in agreement with the QCM‐D results (Figure 3). As in the latter case, the affinity and selectivity increased with increasing χ_E4‐Gal_ (Figure 5B) whereas the overall protein uptake dropped. Once more it was found that the rSAM with χ_E4‐Gal_ = 0.50 displayed the optimum combination of high affinity (K_d_ = 369 ± 50 nM) and capacity (F_max_ = 766 ± 46). To confirm that binding relied on the anticipated ligand receptor interaction we performed two control experiments by 1) comparing binding of LecA with the Fucose/Mannose selective protein LecB and 2) changing the rSAM composition replacing E4‐Gal with the noncomplementary ligand E4‐SA (Figure 5A). The pronounced binding preference for LecA over LecB and the low binding of LecA to the rSAM based on E4‐SA offers adequate proof in support of a recognition driven binding.

Numerous approaches to design multivalent Gal‐presenting receptors for LecA have been published and it is therefore interesting to make a comparison. **Table** **1** lists affinities of the most potent receptors in representative publications comprising various Gal‐ peptide or macrocycle conjugates, Ganglioside presenting supported lipid bilayers, and the example presented in this work. It is clear that potent multivalent receptors can now be prepared by synthetic organic chemistry exploiting peptide or macrocyclic scaffolds. Careful design or high valency numbers involving cumbersome synthetic protocols are here required to reach high affinities. In addition, heterovalency can only be explored by orthogonal conjugation chemistry which often lacks spatial control. Here the adaptable ligand presentations in the form of supported lipid bilayers and rSAMs are interesting due to their enhanced ligand mobility with the latter being especially versatile in terms of preparation and handling.

**Table 1:** Comparison of synthetic β‐Galactose‐based receptors for Lec A.

Lectin binder	Ligand‐scaffold^a)^	Scaffold	Valency^b)^	K_d_ [nM]	Heterovalency^c)^	Comment	Refs.
β‐GalOMe	‐	‐	1	150 000	‐	‐	[46]
Peptide conjugate	covalent	Polyproline	2	112	‐	Distance tuning needed	[23]
Cyclopeptide conjugate	covalent	Cyclic decapeptide	4	22	Orthogonal conjugation	Multistep synthesis Limited ligand mobility	[24]
Macrocycle	covalent	Calix[4]arene	4	420	‐	Multistep synthesis Limited ligand mobility	[21]
Macrocycle	covalent	Calix[6]arene	6	140	‐	Multistep synthesis Limited ligand mobility	[21]
Ganglioside (Gb3)	non‐covalent	Lipid bilayer	n × 1	90	Mixing lipids	Mobile ligands Multistep preparation Poor stability	[47]
E4‐Gal	non‐covalent	rSAM	n × 1	319	Mixing amidines	Mobile ligands, Spontaneous assembly, tunable stability	^d)^

^a)^ Nature of bond between ligand and scaffold.

^b)^ n = number of mono‐ or multivalent ligands attached to the scaffold.

^c)^ Means to prepare heteromultivalent receptors.

^d)^ This work.

### 2.4 rSAMs on Glass Exhibit High Affinity for *PA* and *SG*, in Agreement with the Adhesin Binding Preference

Encouraged by the high affinity of the rSAM for the PA adhesin we turned to test the affinity of the surfaces for this bacterial species. As a reference, we used *Streptococcus gordonii* (SG), a Gram‐positive commensal bacterium prevalent in the skin, oral cavity, and intestine. It easily attaches to host tissues via binding of its adhesins to host cell sialic acids^[^ ^44^ ^]^ or galactose.^[^ ^45^ ^]^

**Figure 6:**
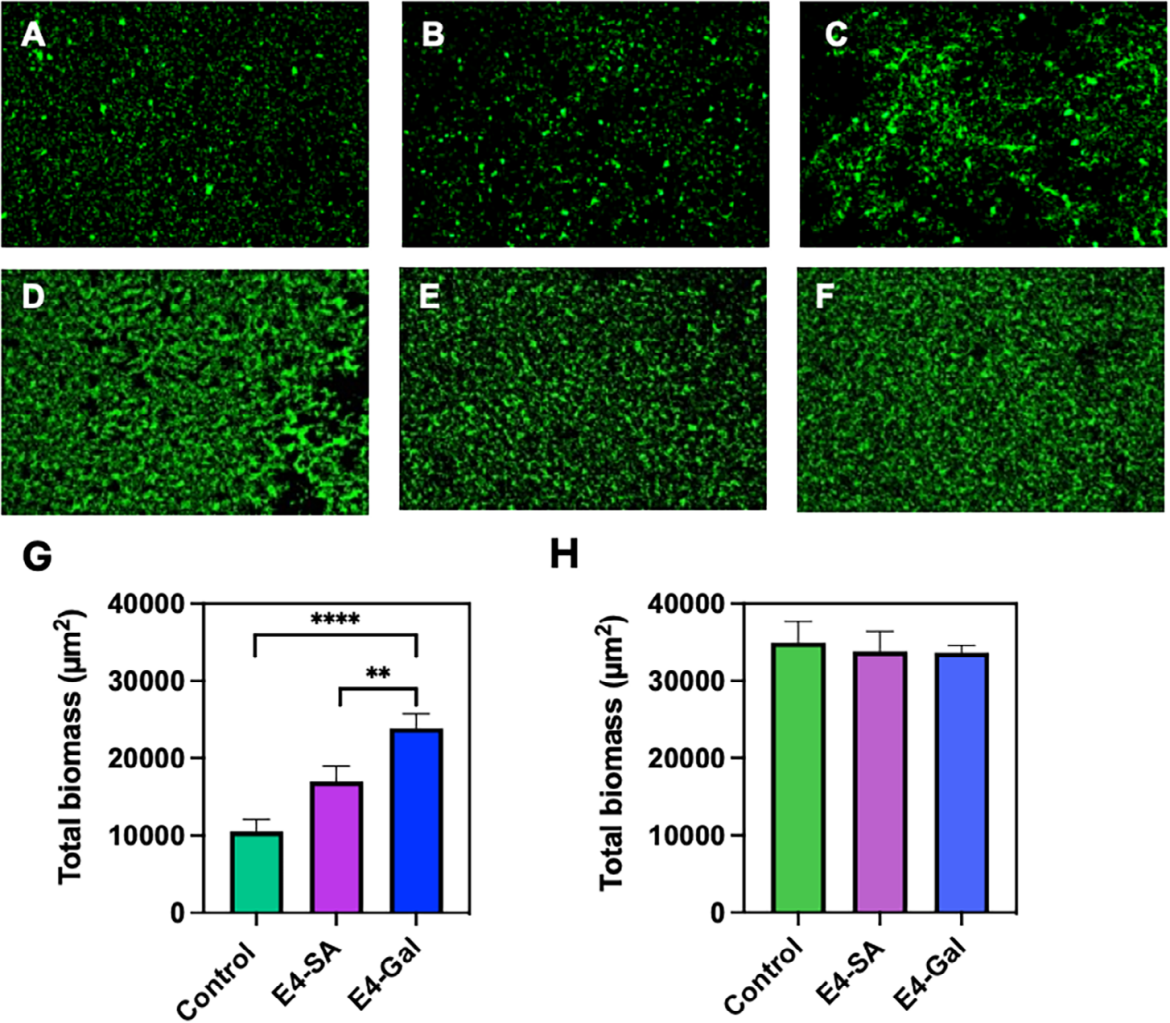
Adhesion of PA01 (A–C) and SG DL1 (D–F) to bare control (A, D), E4‐Gal‐ (C, F) and E4‐SA‐rSAM (B, E) modified glass disks after 2 h of incubation. (G) and (H) show the total biomass of PA (G) and SG (H) estimated as the surface coverage of adhered cells (*n* = 3, p < 0.01 ** or 0.0001 ****).

rSAMs with either χ_E4‐Gal_ = 0.50 or χ_E4‐SA_ = 0.50 were thus prepared and compared with respect to their affinity for the planktonic strain PA01. Glass disks coated with either the E4‐SA rSAM or E4‐Gal rSAM were covered with 150 µl of culture containing approximately 10^8^ CFU/mL bacteria and incubated for 2 hours at 37 °C on the bare glass slides and modified surfaces. The slides were then washed 3 times with phosphate‐buffered saline (PBS) to remove unbound or weakly bound bacteria. Finally, bound bacteria were stained with SYTO 9 dye for 5 minutes and imaged microscopically (**Figure** **6**).

The results show that the surface coverage of adhered PA01 planktonic cells was significantly higher on the E4‐Gal rSAM surface compared to the control surface displaying sialic acid. This was accompanied by an earlier onset of biofilm formation and a significant decrease in motility, the latter contrasting with the behavior of the cells on the unmodified control surface (data not shown). Turning to the reference species SG DL1, there were no significant difference between the number of adhered cells on the E4‐Gal‐ and E4‐SA‐rSAMs, a result in line with this species' adhesin preference for both of the sugars (vide supra).

## 3 Conclusion

This report shows that rSAMs can be used as a multi‐reusable surface functionalization for bacterial adhesin binding and bacterial recognition. These fully restorable surfaces can be conveniently engineered to display high affinity binding of LecA or sialic acid binding lectins exceeding that of their covalently linked counterparts. Experiments conducted on gold and silica substrates using the QCM‐D technique showed that substrate choice does not impact the overall functionality and binding capabilities of the rSAM surfaces. Moreover, a comparative experiment of LecA binding to E4‐Gal surfaces as measured by a multifunctional plate reader showed a similar K_d_ value to that obtained on the QCM‐D, both values in the order of those obtained for several synthetic multivalent LecA receptors. Tests on PA cultures showed that the surface modification led to enhanced adhesion of planktonic cells to E4‐Gal‐rSAM modified surfaces compared to E4‐SA‐rSAM, suggesting a highly versatile approach to engineer surfaces for discrimination and sensing of pathogens. From another aspect, the report highlights the need for convenient approaches to reversibly modify glass and metal oxide surfaces. Multiple processing steps are often required to prepare SAMs on such surfaces (e.g., microscope slides and cover slides) widely used in biology. Restorable surface coatings are scarce, with most examples exploiting dynamic combinatorial chemistry linkages such as disulfide exchange reactions or supramolecular host‐guest interactions.^33^ The approach reported here addresses these problems by allowing fine tuning of surface properties based on one single glass slide. Contrasting with previous reports, the surface functionalization procedure can be readily reversed while offering a way to impart a mobile ligand display. The latter feature is key to boosting binding affinity and selectivity for a given target.

## Conflict of Interest

B.S. and Y.S. are co‐inventors on a patent covering the concept presented in this report. The remaining authors declare no competing interests.

## Supporting information



Supporting Information

## Data Availability

The data that support the findings of this study are available in the supplementary material of this article.
