# Supplementary material for: A Reversible and Dynamic Surface Functionalization for Fluidity Controlled Multivalent Recognition of Lectins and Bacteria
Source: Adv Sci (Weinh). 2025 Apr 26;12(22):2416658. doi: 10.1002/advs.202416658 (PMC12165121; doi:10.1002/advs.202416658)
Supplement: Supplementary file 1 — Supporting Information [file ADVS-12-2416658-s001.pdf]

ADVANCED SCIENCE Open Access

Supporting Information

for *Adv. Sci.*, DOI 10.1002/advs.202416658

A Reversible and Dynamic Surface Functionalization for Fluidity Controlled Multivalent Recognition of Lectins and Bacteria

Thomas Hix-Janssens, Adam Tillo, Hanna Isaieva, Zita Lopes da Silva, Zahra Fatahi, Michele Larocca, Gustav Sedelius, Sara Björk Sigurdardóttir, Yulia Sergeeva, Tiba Al-Dujaili, Julia R. Davies, Kushagr Punyani and Börje Sellergren**

Supplementary Information

A reversible and dynamic surface functionalization for fluidity controlled multivalent recognition of lectins and bacteria

Thomas Hix Janssens,[1] Adam Tillo,[1] Hanna Isaieva,[1] Zita da Silva,[2] Michele Larocca,[1] Gustav Sedelius,[1] Sara Björk Sigurdardóttir,[1] Yulia Sergeeva,[1,3]* Tiba Al-Dujaili,[1] Julia R. Davies[2], Kushagr Punyani[4] and Börje Sellergren[1,3]*

1) Biofilms Research Center for Biointerfaces, Department of Biomedical Science, Faculty of Health and Society, Malmö University, 205 06 Malmö, Sweden
2) Section for Oral Biology and Pathology, Faculty of Odontology, Malmö University, 205 06 Malmö, Sweden
3) Surecapture Technologies AB, Forskaren 1, Per Albin Hanssons väg 35, 21432 Malmö, Sweden.
4) Spermosens AB, The Spark, Medicon Village, Lund, Sweden.

Table of contents

1 Materials and methods 2
1.1 Materials 2
1.2 Instrumentation 2
1.3 Synthesis of 2-7 4
1.4 Surface preparations and QCM-D measurements 6
1.5 Surface preparations and fluorescence measurements 7
1.6 Bacterial attachment and biomass quantification 8
2 Tables and figures 10
2.1 Water contact angle (WCA) 10
2.2 Infrared reflection absorption spectroscopy (IRAS 11
2.3 Quartz crystal microbalance with dissipation monitoring (QCM-D) 16
2.4 Fluorescence measurements on modified glass slides with LecA 18
2.5 Evidence supporting the identity of new compounds 20

1 Materials and methods

1.1 Materials

3-Butyn-1-ol was purchased from Sigma-Aldrich (Merck kGaA) and stored with freshly activated 3Å molecular sieves before use. Boron trifluoride etherate ($BF_3 \cdot Et_2O$) was purchased in 5 mL bottles from Sigma-Aldrich (Merck kGaA) and only used within a month of first opening a new bottle. Extra dry dichloromethane, 99.9% AcroSeal™, stabilized with 50 ppm amylene, was purchased from Thermo Fisher Scientific. Sodium methoxide (NaOMe) in methanol (5M) was purchased from Tokyo Chemical Industry (TCI). β-D-galactose pentaacetate was purchased from FluoroChem Ltd. All other reagents were purchased from Sigma-Aldrich (Merck kGaA) in standard quality and used without purification. The filler amidine E2-OH and the sialic acid (SA) terminated amidine E4-SA were synthesised as described in our previous reports.[1-3] Human serum albumin (HSA) came from Sigma-Aldrich (Merck kGaA). FITC-labeled LecA and LecB were purchased from Elicityl (Crolles, France).

1.2 Instrumentation

Fourier-transform infrared spectroscopy (FTIR) was performed using a Nicolet 6400, equipped with a liquid nitrogen cooled MCT-A detector or a DTGS detector. The *smartSAGA* accessory (angle of incidence 80°) was used to collect the data for reflective gold surfaces at resolution 4 for a total of 250 scans. For the transparent modified glass slides, the *smartiTR* accessory was used. 1000 spectra were collected at resolution 4. Dried compressed air was continuously run through the instrument during and before the measurements. Correction of the baseline and evaluation of data was performed using the OMNIC 6 software.

Water contact angle (WCA) measurements were used to detect changes in surface hydrophilicity and were performed using a Drop Shape Analyzer 100 (Krüss). Ultrafiltered water was used for the analysis and three measurements per surface were taken to provide statistical mean and standard error.

Quartz crystal microbalance with dissipation monitoring (QCM-D) was performed using a QSense E4 system (Biolin Scientific, Sweden). Frequency shifts (Δf_n) and changes in the dissipation (ΔD) were monitored over different overtones on both silica-coated and gold-coated quartz sensor chips. When solutions were run through the modules, the flow rate was set to 100 µL/min using an Ismatec peristaltic IPC-N4 pump. The pump was turned off in between

injections for 20 minutes to let the system equilibrate. All experiments were performed in 10 mM HEPES buffer at pH 8 and at a constant temperature of 25 °C. Data was plotted using the QTools software (Biolin Scientific, Sweden). Sauerbrey modelling was done using equation 1.

$$\Delta f_n = -\frac{2f_0 m_f}{Z_q} \quad \text{(eq. 1)}$$

Where Δf_n equals the frequency change of a specific overtone n, f_0 is the fundamental resonance frequency of a quartz sensor (~ 5 MHz), Z_q is the acoustic impedance of quartz (8.8×10^6 kg m^{-2} s^{-1}), and finally m_f is the mass of the thin film per unit area (kg m^{-2}). Thus, the adsorbed mass m_f can easily be calculated from the data, provided it follows Sauerbrey conditions. These are met when (i) the added mass onto the surface is small relative to the mass of the crystal, (ii) a rigid layer is present, and (iii) when the mass is evenly distributed over the surface. In general, these can be identified by overlapping Δf_n of the overtones, together with minimal changes in ΔD.

Fluorescence measurements were conducted using a Tecan Safire microplate reader (Tecan, Switzerland). Fluorescence intensity was measured using bottom-reading mode. The gain was set to a constant value of 100. For top-reading mode, the gain and Z-position were optimized for the control solution with the highest concentration. The excitation wavelength was set to 495 nm (LecA, LecB) or 400 nm (HSA) with slit width 5 nm, and the emission wavelength was set to 519 nm (LecA, LecB) or 510 nm (HSA) with slit width 7.5 nm.

Nuclear magnetic resonance (NMR) spectra were recorded using an Agilent (Varian) Mercury 400 MHz spectrometer at 25°C using deuterated chloroform ($CDCl_3$) or methanol (CD_3OD). The spectra were processed and analyzed using MestReNova or Bruker TopSpin 4.3.0 software, using the solvent residual peaks as a reference. NMR data is presented as chemical shift in ppm (multiplicity [s, singlet; d, duplet; t, triplet; q, quartet], coupling constant(s) *J*, number of protons).

Mass spectrometry was performed by direct injection of samples dissolved in water and methanol into a Waters Micromass ZQ detector operating in the electrospray ionization mode (ESI).

1.3 Synthesis of 2-7

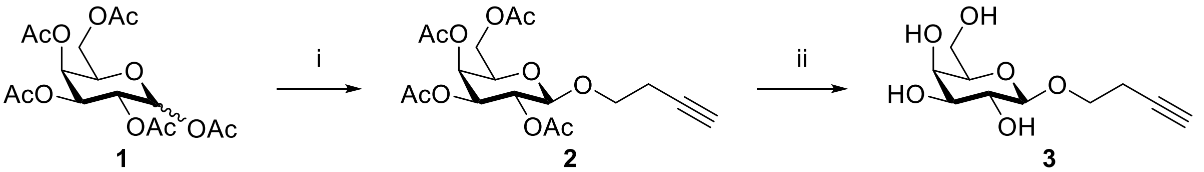

Scheme S1. Synthesis of **3** from galactose pentaacetate. *Reagents and conditions:* i) 3-butyn-1-ol, $BF_3 \cdot Et_2O$, dry DCM, 0°C to r.t., 1 day; ii) NaOMe, MeOH, r.t., 1.15 h.

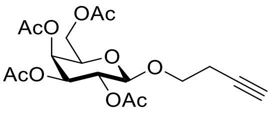

*(3-butyn-1-yl) 2,3,4,6-tetra-O-acetyl-β-D-galactopyranoside (**2**)*

Under a nitrogen atmosphere, to β-D-galactose pentaacetate **1** (600 mg, 1.54 mmol) in dry dichloromethane (6 mL) was added 3-butyn-1-ol (223 µL, 3.08 mmol, 2 equiv.) and the resulting mixture cooled in an ice-water bath before adding $BF_3 \cdot Et_2O$ (380 µL, 3.08 mmol, 2 equiv.). The mixture was allowed to warm to room temperature, stirred for 5 hours and another portion of $BF_3 \cdot Et_2O$ (190 µL, 1.54 mmol, 1 equiv.) added. After stirring for another 12 hours at room temperature, the organic phase was washed with saturated $NaHCO_3$ (9 mL), the aqueous phase extracted with dichloromethane (15 mL), the combined organic phases dried over $MgSO_4$, and the solvent removed *in vacuo*. Purification by flash column chromatography (EtOAc:n-heptane 8:2) afforded the product as a yellowish oil (566 mg, 92%), which was used without further purification. Calc. mass $[M+Na]^+$ 255.08; found mass $[M+Na]^+$ 255.95 (ESI^+). ^1H NMR (400 MHz, CD_3OD) δ 5.38 (d, J = 2.9 Hz, 1H), 5.10 (d, J = 33.6 Hz, 1H), 4.85 (s, 1H), 4.69 (d, J = 7.1 Hz, 1H), 4.13 (d, J = 26.7 Hz, 4H), 3.89 (td, J = 6.5, 9.9 Hz, 1H), 3.67 (td, J = 7.0, 9.9 Hz, 1H), 3.31 (s, 1H), 2.51 (d, J = 16.1 Hz, 1H), 2.45 (d, J = 18.2 Hz, 2H), 2.15 (s, 3H), 2.05 (s, 3H), 2.03 (s, 3H), 1.94 (s, 3H) ppm.

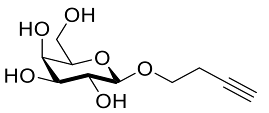

*(3-butyn-1-yl) β-D-galactopyranoside (**3**)*

Compound **2** (566 mg, 1.41 mmol) was dissolved in methanol (11 mL) and a solution of NaOMe in methanol (5M, 142 µL, 0.71 mmol, 0.5 equiv.). The resulting mixture was stirred at room temperature for 1.15 h before adding an ion exchange resin (Amberlite IR-120 H^+, 100 mg) to neutralize the solution. After stirring for 5 minutes, the resin was filtered off and the solvent removed *in vacuo* to afford the product as a brown oil (320 mg, 98%). Calc. mass $[M+Na]^+$ 255.08; found mass $[M+Na]^+$ 255.95 (ESI^+). ^1H NMR (400 MHz, $CDCl_3$) δ ^1H NMR (400 MHz, cd_3od) δ ^1H NMR (400 MHz, cd_3od) δ 4.25 (d, *J* = 7.4 Hz, 1H), 3.95 (td, *J* = 7.4, 9.6 Hz, 1H), 3.82 (d, *J* = 2.4 Hz, 1H), 3.72 (m, 4H), 3.63 (t, *J* = 6.9 Hz, 1H), 3.51 (m, 2H), 3.46 (dd, *J* = 3.2, 9.7 Hz, 1H), 3.31 (s, 1H), 2.53 (t, *J* = 3.6 Hz, 1H), 2.51 (t, *J* = 7.3 Hz, 2H) ppm.

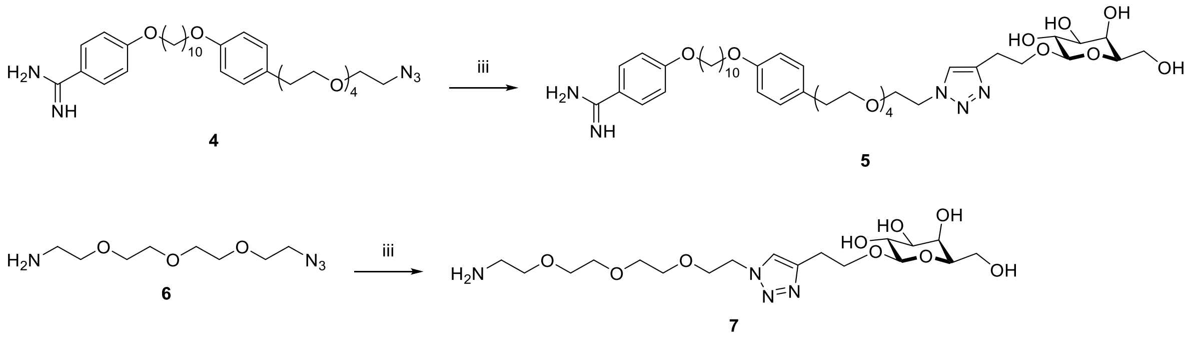

Scheme S2. Click coupling of **3** to amidine **4** (synthesized as previously reported [1-2]) and amino azide **6**. *Reagents and conditions:* iii) Sodium ascorbate, $CuSO_4$, H_2O/MeOH, r.t., 1 day.

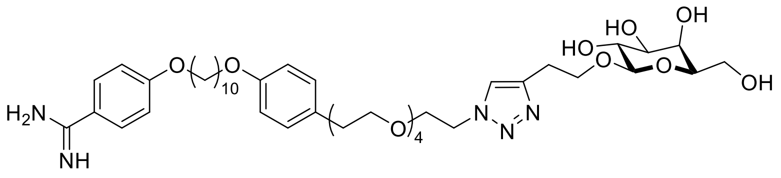

5

4-((10-(4-(14-(4-(2-(β-D-galactopyranoside-1-yloxy)ethyl)-1H-1,2,3-triazol-1-yl)-3,6,9,12-tetraoxatetradecyl)phenoxy)decyl)oxy)benzamidine trifluoroacetate ***(5, E4-Gal)***

Solution of sodium ascorbate (0.237 g, 1.2 mmol) and $CuSO_4$ (0.150 g, 0.6 mmol) in water (3 mL) was added dropwise to a vigorously stirred alkyne galactose **3** (0.278 g, 1.2 mmol) and compound **4** (0.780 g, 1.2 mmol) in 6 mL of MeOH. Formed dense, purple mixture was diluted with additional 4 mL of 50% MeOH, sonicated and stirred overnight at room temperature. Next, the reaction mixture was concentrated, diluted with brine and extracted three times with THF and MeCN. Combined organic layers were dried with $MgSO_4$, vaporized to dryness, and purified on C-18 silica gel column (MeCN/H_2O 10 to 100% + 0.1% TFA) to give compound **5**

(E4-Gal) as an off-white powder (0.337 g, 29% yield). R_f (DCM/MeOH 3 : 1) 0.40. $[\alpha]_D^{20}$ = -13.4 (c = 0.5, MeOH). Calc. mass $[M+H]^+$ 847.02; found mass $[M+H]^+$ 847.58 (ESI^+). ^1H NMR (400 MHz, CD_3OD) δ 7.99 (s, 1H), 7.84 (d, *J* = 9.2 Hz, 2H), 7.11 (d, *J* = 8.8 Hz, 2H), 6.96 (d, *J* = 8.8 Hz, 2H), 6.81 (d, *J* = 8.8 Hz, 2H), 4.54 (t, *J* = 5.2 Hz, 2H), 4.26 (d, *J* = 7.6 Hz ,1H), 4.01 (t, *J* = 6.4 Hz, 2H), 3.93 – 3.84 (m, 5H), 3.74 (m, 2H), 3.63 (t, *J* = 6.8 Hz, 2H), 3.58 (m, 18H), 3.51 (m, 3H), 3.35 (s, 2H), 3.03 (t, *J* = 6.0 Hz, 2H), 2.77 (t, *J* = 7.2 Hz, 2H), 1.76 (m, 4H), 1.46 (m, 4H), 1.35 (m, 12H) ppm. ^{13}C NMR (101 MHz, CD_3OD) δ 163.6, 159.0, 132.1, 130.9, 130.6, 125.5, 115.4, 115.2, 105.0, 76.6, 74.9, 73.5, 72.5, 71.5, 71.4 (2), 71.2, 70.3 (2), 69.3, 69.2, 69.0, 62.5, 51.6, 49.8, 36.2, 30.6, 30.4 (2), 30.2, 27.1 (2) ppm.

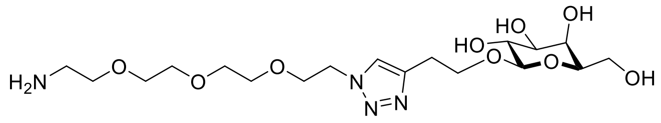

7

Synthesis of 2-(2-(2-(2-(4-(2-(β-D-galactopyranoside-1-yloxy)ethyl)-1H-1,2,3-triazol-1-yl)ethoxy)ethoxy)ethoxy)ethanamine (7)

Solution of sodium ascorbate (0.128 g, 0.65 mmol) and CuSO4 (0.081 g, 0.32 mmol) in water (1 mL) was added dropwise to a vigorously stirred alkyne galactose **3** (0.150 g, 0.65 mmol) and 11-Azido-3,6,9-trioxaundecan-1-amine (130 µL, 0.65 mmol) in 2mL of MeOH. Reaction mixture was sonicated and continued at room temperature overnight. Obtained suspension was evaporated to dryness, reconstituted in MeOH, and centrifuged. Solution was decanted, concentrated, and purified by means of column chromatography (SiO_2, EtOH/H_2O 8:2 to 1:1 + 0.5% TEA) to give compound **7** as a yellow oil (0.123 g, 42% yield). R_f (EtOH/H_2O 3:1 + 0.5% TEA, spots visualized with 10% H_2SO_4 in EtOH) 0.28. $[\alpha]_D^{20}$ = -16.2 (c = 0.5, MeOH).). Calc. mass $[M+H]^+$ 451.49; found mass $[M+H]^+$ 451.86 (ESI^+). ^1H NMR (400 MHz, CD_3OD) δ 7.97 (s, 1H), 4.56 (t, *J* = 4.8 Hz, 2H), 4.29 (d, *J* = 6.4 Hz, 1H), 4.14 (m, 1H), 3.90 – 3.83 (m, 4H), 3.73 (m, 4H), 3.61 (m, 8H), 3.52 (m, 4H), 3.35 (s, 1H), 3.16 (m, 2H), 3.02 (t, *J* = 6.4 Hz, 2H), 1.32 (m, 2H) ppm. ^{13}C NMR (101 MHz, CD_3OD) δ 146.2, 125.1, 105.0, 76.7, 75.0, 72.5, 71.5, 71.3 (2), 71.2, 70.4, 70.2, 69.4, 68.0, 62.5, 51.3, 40.7, 27.2 ppm.

1.4 Surface preparations and QCM-D measurements

Prior to modification with the anchor SAM, both gold and quartz sensor chips were plasma cleaned for 5 minutes. Cleaned quartz sensor chips were then modified for 18h with 3-aminopropyldimethylethoxysilane (ADMES; 10% v/v) in ethanol. After rinsing the surface

with MilliQ water, they were subsequently modified for 4h with succinic anhydride (4 w/v%) in a solution containing THF and TEA (95:5). The cleaned gold sensor chips on the other hand were modified for 18h with 20 µM mercaptohexadecanoic acid (MHA) in an ethanol solution containing 10% v/v acetic acid. Preparation of the rSAM layer was performed *in situ* by flowing the amidine solutions (total concentration 50 µM) containing different ratios of the galactose-bearing amidine (E4-Gal) and the filler E2-OH for 10 min at a flow rate of 0.1 mL/min. The flow was then stopped and the system allowed to equilibrate for 30 min followed by rinsing with HEPES buffer for 10 min at a flow rate of 0.1 mL/min. The flow was again stopped and the system allowed to equilibrate until stable baseline, the flow restarted before injecting protein solutions (see section 1.2). Surfaces containing covalently attached β-Gal were prepared *ex situ* by EDC/NHS catalysed coupling of galactose-appended amine (**7**) to an MHA-SAM using ethylamine as filler. Prior to preparation of the surfaces bearing covalently attached β-Gal, the sensors were rinsed with trimethylamine solution (10% v/v in ethanol) and activated with EDC/NHS mixture (200 mM and 50 mM respective solutions in water, added as 1:1 vol mixture and incubated for 15 min). Surfaces were subsequently washed with deionized water and reacted with galactose-appended amine **7** (50 µM solution in PBS pH=8 with 50 µM ethylamine as filler) over 1.5 h, at room temperature. After obtaining stable f and D values, and rinsing with 10 mM HEPES buffer pH 8, the sensors were investigated for their response to solutions of increasing concentrations of proteins, analogously to the experiment described above for the rSAM surfaces.

1.5 Surface preparations and fluorescence measurements

Glass cover slips (Ø 5 mm, Menzel-Gläser) were used as the substrate and were placed in a plasma cleaner (high intensity) for 5 minutes. The activated slips were immersed overnight in an ethanolic solution containing 3-aminopropyldimethylethoxysilane (ADMES; 10% v/v) at room temperature. They were then rinsed with double distilled water and dried under nitrogen after which they were baked in an oven at 70 °C for one hour. Next, the amino-bearing glass cover slips were immersed in a solution containing succinic anhydride (4% w/v) in tetrahydrofuran (THF) with 5% v/v triethylamine for 4 hours at room temperature. They were then rinsed with double distilled water and dried under nitrogen flow. The glass cover slips with finished anchor layers were then placed in a polystyrene 96-well plate with flat bottom (Greiner Bio-One, Germany). Solutions containing a different mole-fractions of E4-Gal (χ=0.05 to

χ=0.50) in 10 mM HEPES buffer (pH 8) were prepared and 130 μL of the solutions were added onto the anchor layer glass cover slips and left to self-assemble overnight at room temperature. The wells were then rinsed three times with HEPES buffer to wash out excess amidines. Protein solutions in the concentration range 0-960 nM were prepared in HEPES buffer. 225 μL of these solutions were then added to wells, the plate was sealed and placed in an orbital shaker for one hour at 4 °C at a shaking speed of 400 rpm. The slips were then rinsed three times with HEPES buffer to wash out weakly bound lectins. The fluorescence of the glass slips was then measured by bottom-mode reading (see section 1.2) with the gain set to 100 and compared with a corresponding reading prior to protein incubation and after surface regeneration (see below). After completion of the protein incubation and fluorescent readings, the cover slips were regenerated by first rinsing with 10 mM Gly-HCl buffer (pH 2). Then, buffer was added and the plate placed on a shaker for 15 minutes at room temperature. This treatment was repeated twice. Finally, the wells were rinsed three times with HEPES buffer.

1.6 Bacterial attachment and biomass quantification

Cultures of *P. aeruginosa* strain PA01 and *S. gordonii* DL1 were stored in 10% skimmed milk at -80°C. For use, bacteria were streaked onto BHI agar and maintained at 37°C, in 5% CO_2 in air for 24 hours. Colonies were transferred into BHI broth and grown overnight at 37°C, in 5% CO_2 in air. Overnight cultures were diluted to grow till OD_{600} = 0.2. An aliquot was then centrifuged (3500rpm, 10 mins) and the pellet resuspended in the same volume of Mueller Hinton broth. Glass disks coated either with the E4-SA or E4-Gal rSAM were covered on one-side with 150 μl of culture containing approximately 10^8 CFU/mL bacteria and incubated for 2 hours at 37 °C on the bare glass or gold slides and modified surfaces. The slides were then washed 3 times with phosphate-buffer saline (PBS) to remove unbound or loosely bound bacteria. Bacterial biomass was then stained with the Syto 9 component of the LIVE/DEAD™ Bac Light™ Bacterial Viability Kit (Invitrogen). Visualization was performed using an OLYMPUS BX 60 microscope, magnification x40, and images of ten randomly selected areas of each sample surface were taken for analysis. Total biomass was quantified using bioImageL v.2.0, and mean surface coverage ± sem was calculated using GraphPad Prism version 10.0.3 (275). All experiments were performed in triplicate using independent biological replicates.

1.7 Statistical analysis

The data reported in Figure 5B, 6, S8 and Table S3 are expressed as mean values ± SD of three independent experiments (n=3) with significance assessed using the t-test.

References

[1] S. Y. Yeung, A. Mucha, R. Deshmukh, M. Boutrus, T. Arnebrant, B. Sellergren, *ACS Central Science* **2017**, *3*, 1198-1207.

[2] S. Y. Yeung, T. Ederth, G. Pan, J. Cicėnaitė, M. Cárdenas, T. Arnebrant, B. Sellergren, *Langmuir* **2018**, *3*, 4107-4115.

[3] S. Y. Yeung, Y. Sergeeva, T. Dam, P. Jonsson, G. Pan, V. Chaturvedi, B. Sellergren, *Langmuir* **2019**, *35*, 8174-8181.

2 Tables and figures

2.1 Water contact angle (WCA)

Table S1. Advancing water contact angles for modified gold surfaces

	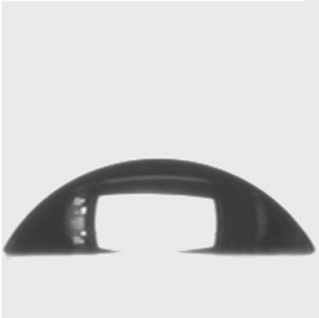	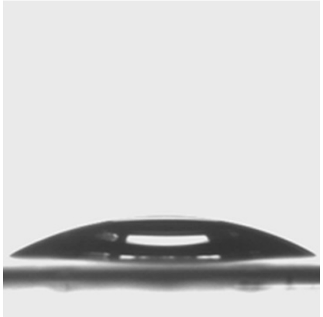	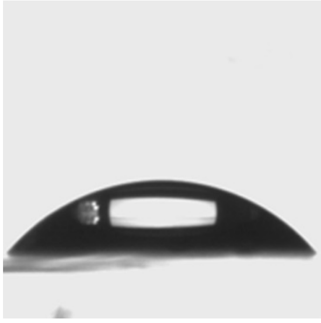
**Surface**	**Au**	**Au-MHA**	**E2-OH**
**Contact angle Θ (°)**	66.5 ± 2.6	32.4 ± 2.7	51.9 ± 0.8
	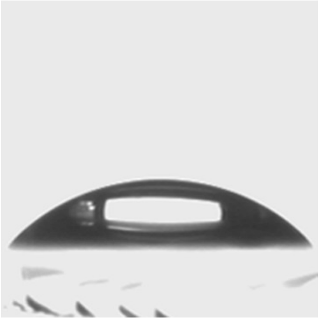	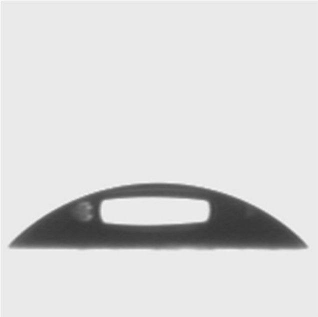	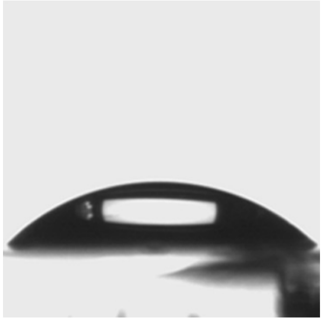
**Surface**	**βGal (25%)**	**βGal (50%)**	**βGal (75%)**
**Contact angle Θ (°)**	48.8 ± 0.8	47.1 ± 1.1	45.2 ± 1.3

2.2 Infrared reflection absorption spectroscopy (IRAS

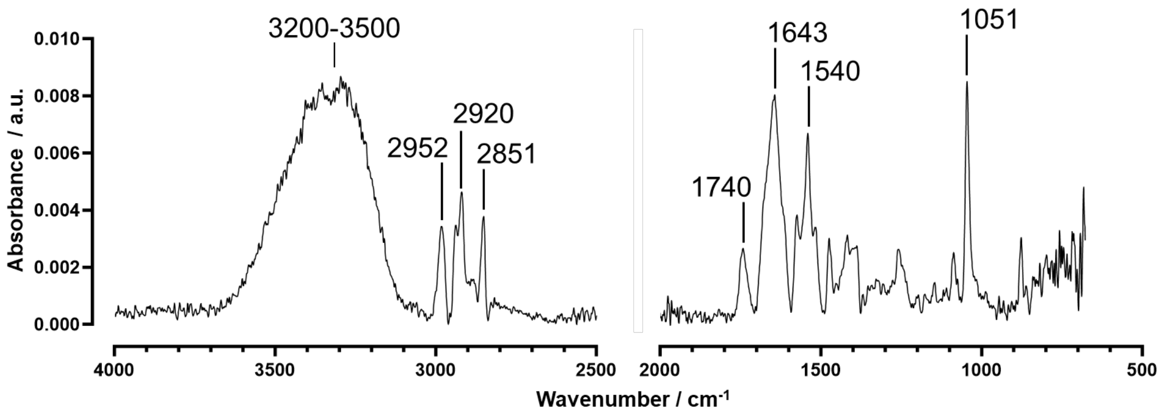

Figure S1. Baseline corrected attenuated total reflection (ATR) IR absorption spectrum (1000 scans, resolution 4) of E4-Gal in solution.

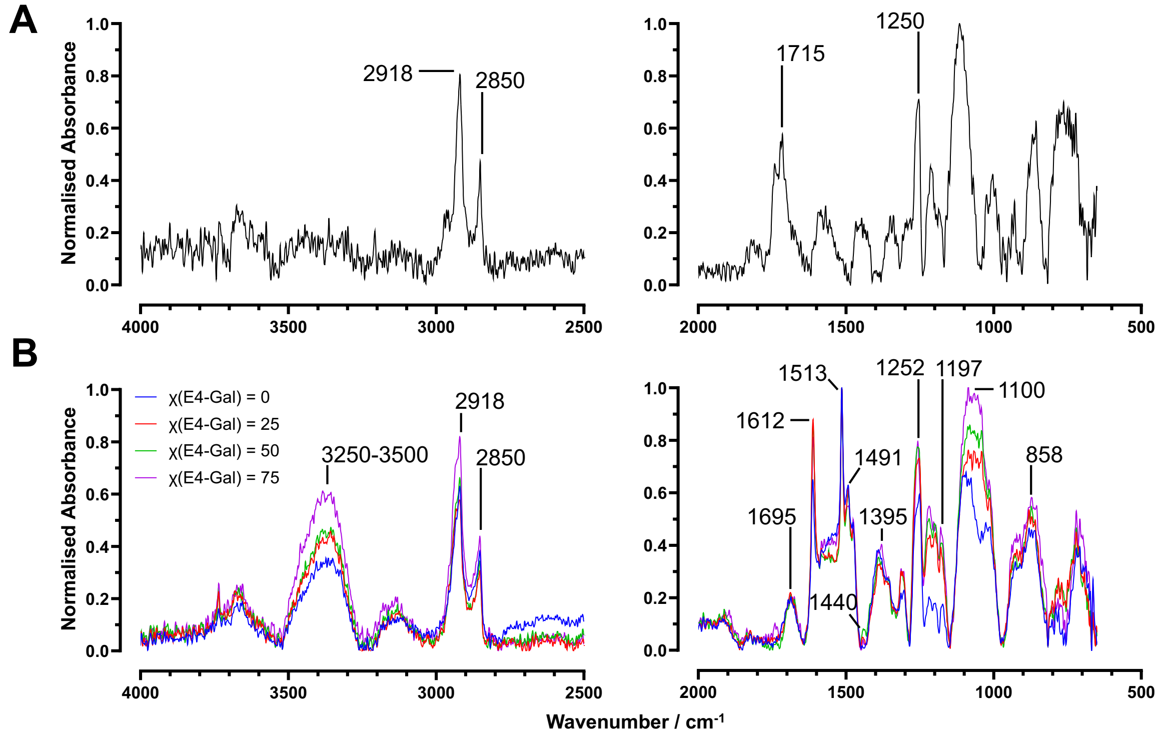

Figure S2. Baseline corrected IRAS-spectra (1000 scans, resolution 4) of A) the MHA anchor SAM on gold and B) overlays of the mixed rSAMs of E2-OH/E4-Gal with $\chi_{E4\text{-}Gal}$ = 0 (blue); 0.25 (red); 0.50 (green); 0.75 (purple).

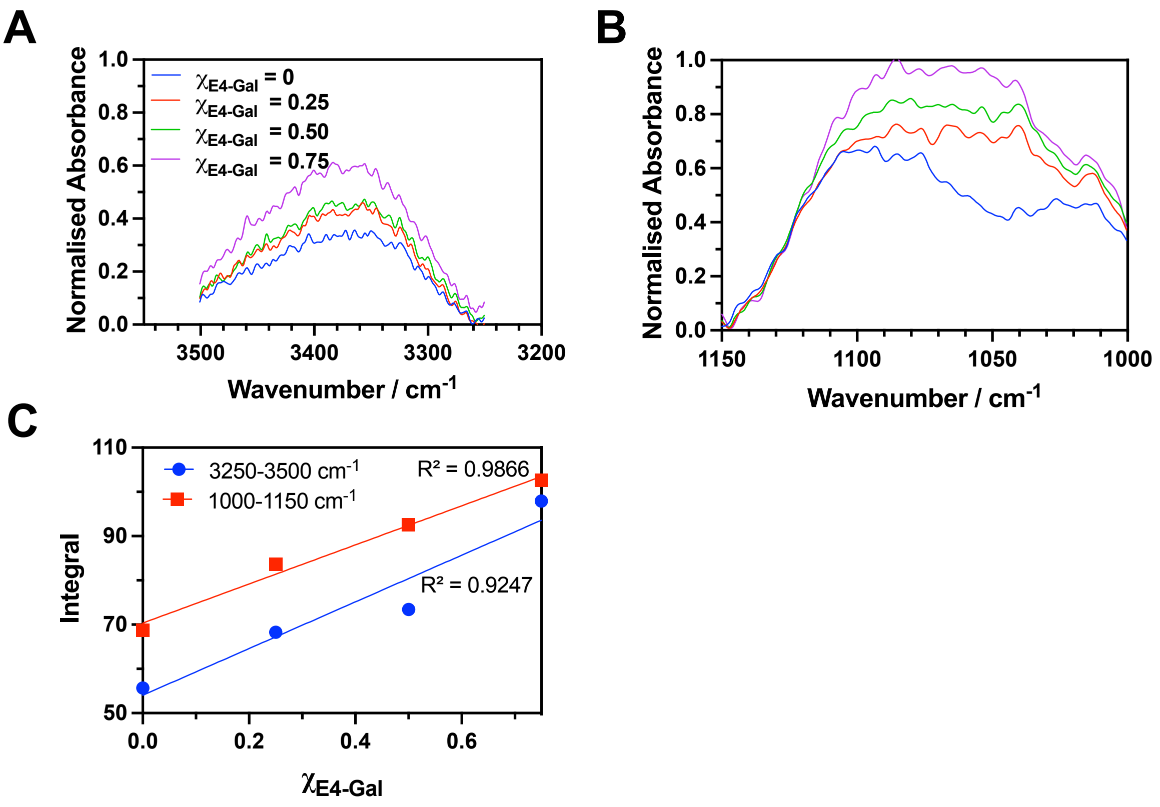

Figure S3. Magnified regions of the spectra in Fig. S2 corresponding to (A) the NH/OH stretch 3250-3500 cm-1 band and (B) the 1000-1150 cm-1 ether C-O-C stretch band. In (C) the band integrals have been plotted against $\chi_{E4\text{-}Gal}$ and fitted to straight lines.

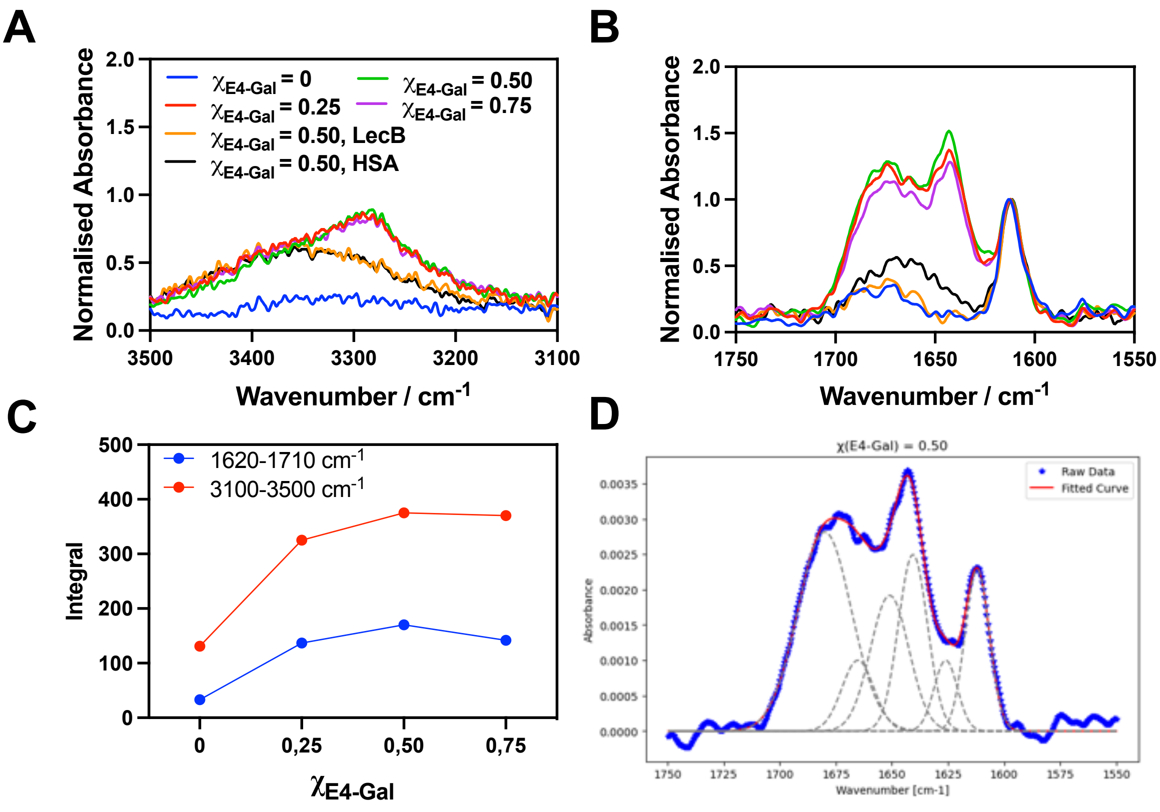

Figure S4. Baseline corrected IRAS-spectra (1000 scans, resolution 4) of the mixed rSAMs in Fig. S2 after incubation in protein solutions of LecA, LecB and HSA (500 nM in HEPES buffer pH 8) followed by rinsing with HEPES buffer. A and B show the bands corresponding to the NH and OH stretch at 3100-3500 cm^{-1} and the amide I band at 1620-1710 cm^{-1} respectively. C shows the integrals of the NH/OH stretch (red circles) and amide I (blue circles) bands versus rSAM nominal composition. D shows the deconvolution of the amide I band ($\chi_{E4\text{-}Gal}$ = 0.50) by Gaussian curve fitting for estimating the relative contribution of the different secondary structures.

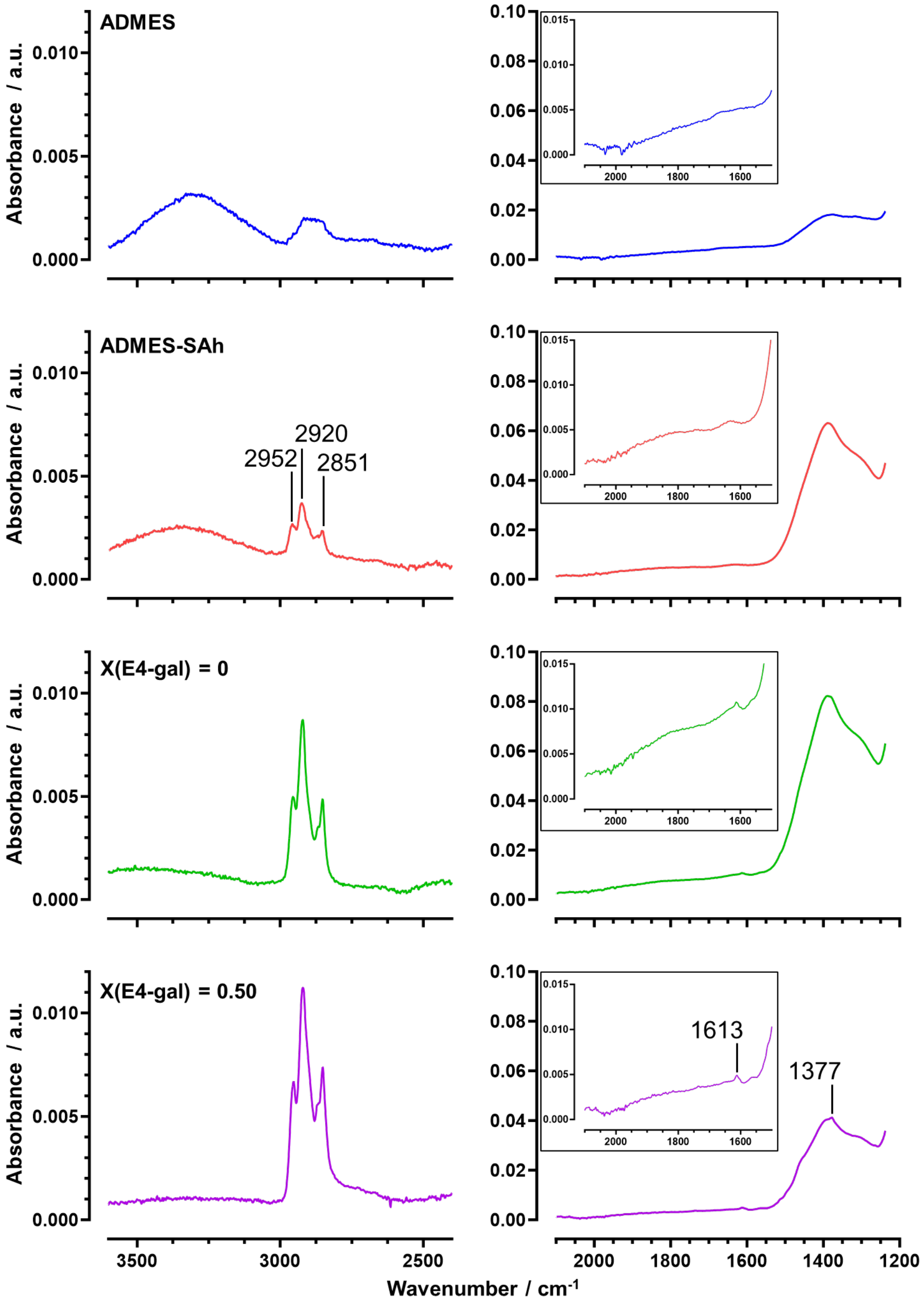

Figure S5. Baseline corrected attenuated total reflection (ATR) IR absorption spectra (1000 scans, resolution 4) of glass slides modified with from top to bottom ADMES (blue), succinic anhydride (red) and rSAMs with $\chi_{E4\text{-}Gal}$ = 0 (green) and 0.50 (purple). The band corresponding to the benzamidinium $(C{=}C)_{1,4}$ stretch vibration at 1613 cm^{-1} has been indicated.

Table S2. Assignment of bands belonging to the spectrum of E4-Gal in Fig. S2.

Wavenumber (cm^{-1})	Band
3200-3500	NH_2, N-H stretch; COOH, O-H stretch
2952	Benzene, C-H stretch
2920	Alkyl, CH_2, C–H stretch (asym)
2851	Alkyl, CH_2, C–H stretch (sym)
1740	Amidinium, N-C=N stretch (asym)
1643	Benzamidine (C=C, C-N)
1540	Benzene C=C stretch (‖1,4 axis)
1250	Aromatic ethers, aryl-O-CH_2- stretch (asym)
1051	Aliphatic ethers, CH_2, C-H bend

Table S3. Results from the Gaussian curve fitting shown in Fig. S4D for estimating the relative contribution of the different LecA secondary structures.

	β-sheet	Random coil	β-turn	C=C ring stretch
**Center peak position (cm^{-1})**	1626, 1640, 1680	1650	1665	1612
**Integral (%)**	60.46	18.03	7.87	13.64

2.3 Quartz crystal microbalance with dissipation monitoring (QCM-D)

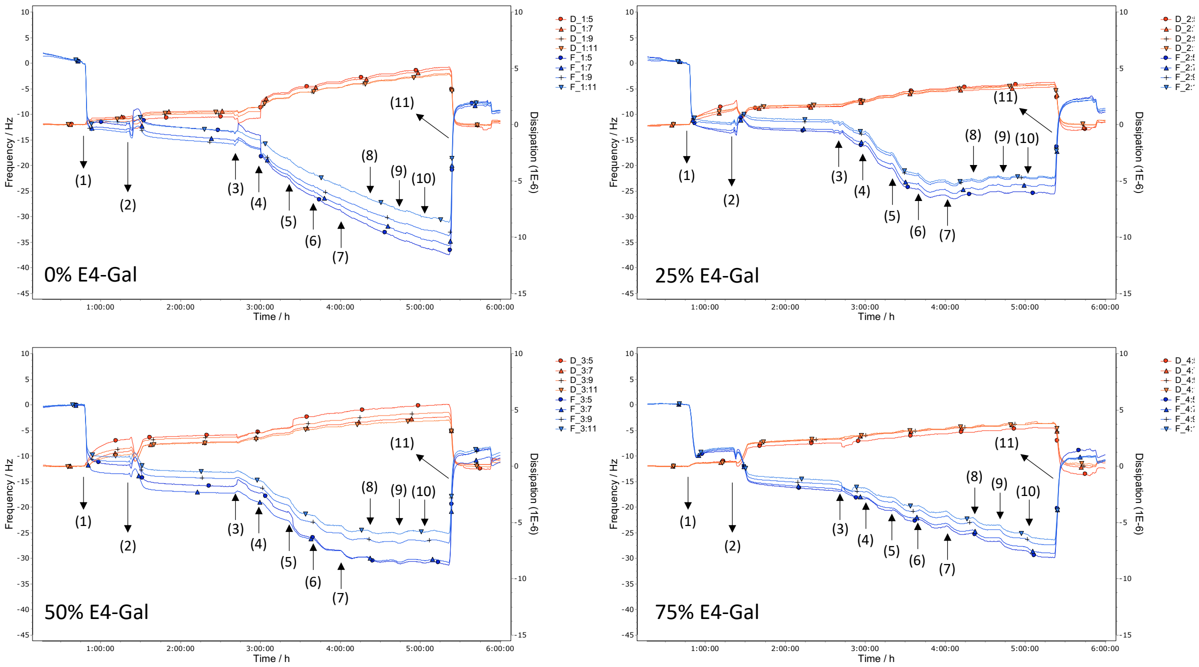

Figure S6. Titration of the galactose-presenting rSAMs with increasing concentrations of LecA. The outer layer is composed of amphiphiles E2-OH and E4-Gal at different molar ratios. Blue and orange plots follow changes in the frequency and dissipation of the lower overtones during the QCM-D experiment, respectively. Black arrows represent: (1) – injection of amidines, (2) – rinsing of the surface with 10 mM HEPES buffer pH = 8.0, (3-10) – titration with aliquots of lectin LecA (60, 120, 180, 240, 360, 480, 720, 960 nM), (11) – disassembly of the rSAM layer with 10 mM Gly-HCl buffer pH = 2.0.

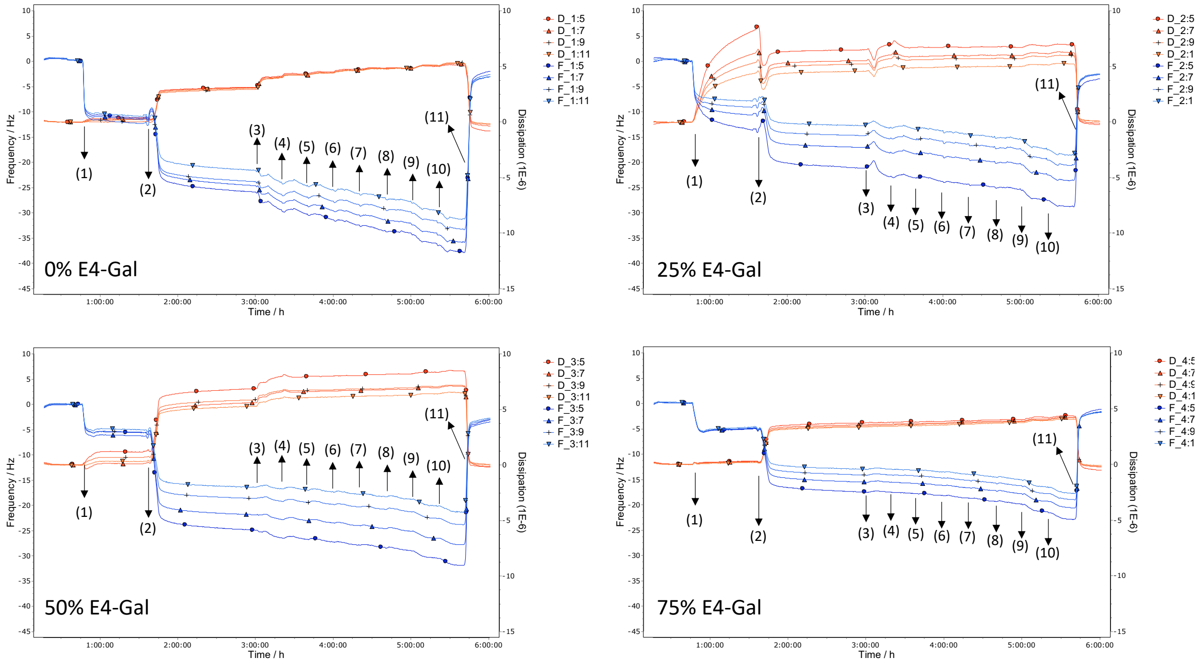

Figure S7. Titration of the galactose-presenting rSAM surfaces on gold substrate with increasing concentrations of HSA. Experimental conditions as described in Fig. S6.

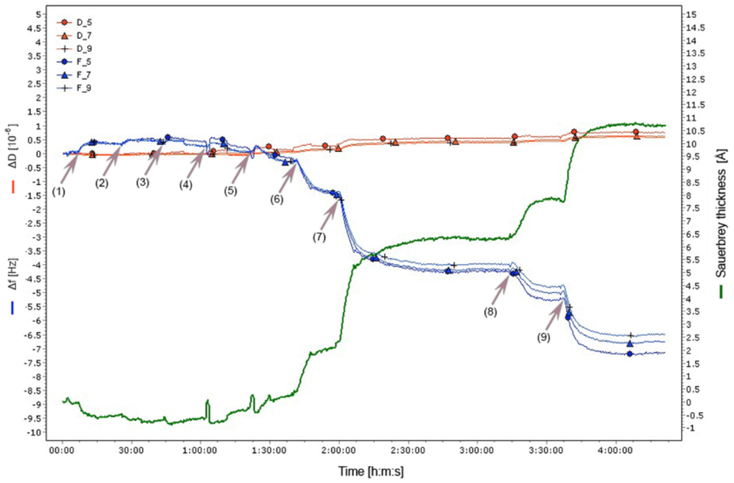

Figure S8. Changes in the frequency and dissipation during titration of the galactose – presenting SAMs with increasing concentrations of LecA. The outer layer is composed of covalently attached **7** and ethanolamine (χ_7 = 0.5). Purple arrows represent titration with aliquots of LecA (15.6, 31.2, 62.5, 125, 250, 500, 1000, 1227, 1840 nM).

2.4 Fluorescence measurements on modified glass slides with LecA

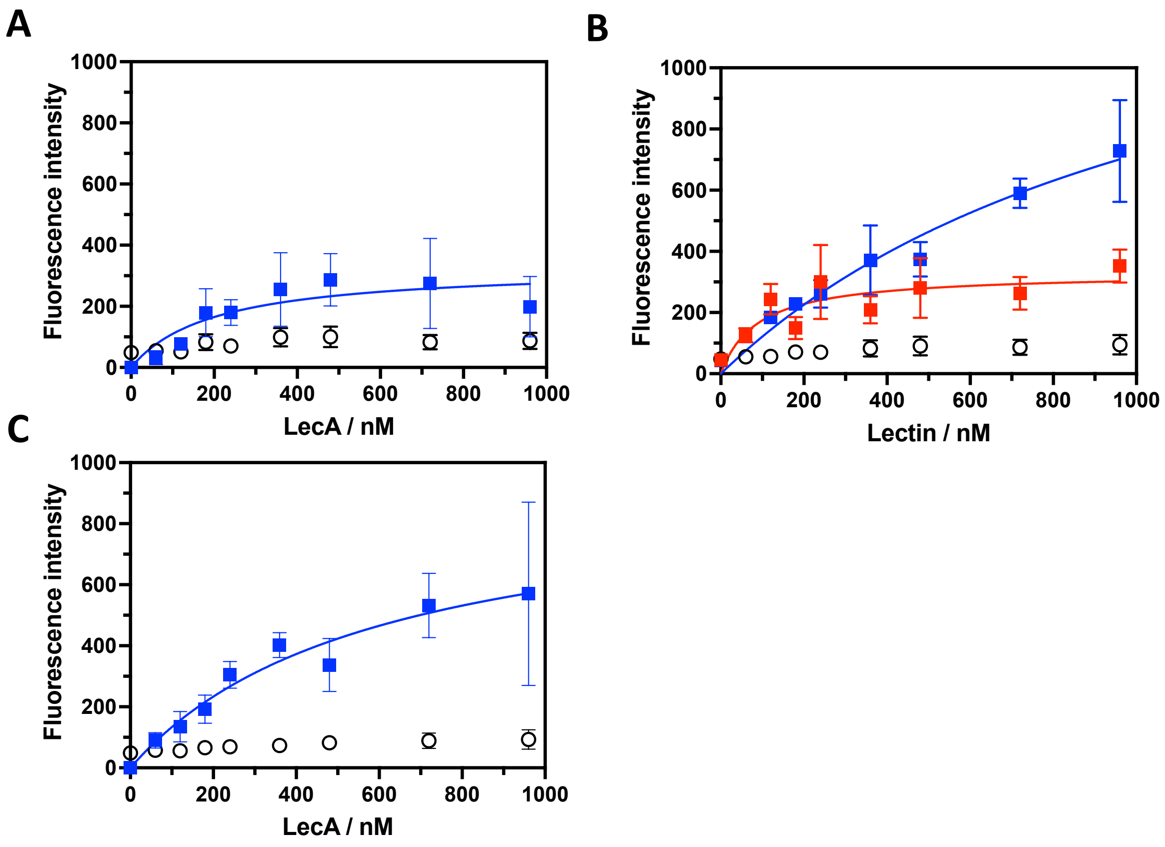

Figure S9. Bottom mode fluorescence measurements of rSAM modified glass cover slips after overnight incubation with amidine solutions containing (A) $\chi_{E4\text{-}Gal}$=0.05, (B) $\chi_{E4\text{-}Gal}$=0.15, (C) $\chi_{E4\text{-}Gal}$=0.25 followed by incubation with LecA (blue curves) or LecB (red curve). Residual fluorescence was checked after rinsing with acidic buffer (open circles). λ_{ex} = 495 nm, slit width 5 nm; λ_{em} = 519 nm, slit width 7.5 nm. Results show average with standard deviation from three independent repeats (n=3).

Table S4. Dissociation constant (K_d) and curve fitting parameters for the adsorption isotherms in Fig 5 and Fig. S9.

rSAM	$\chi_{E4\text{-}Gal(SA)}$	Protein	K_d (nM)	F_{max}	R^2
E4-Gal	0.05	LecA	201±114	330±66	0.829
E4-Gal	0.15	LecA	1196±455	1575±395	0.964
E4-Gal	0.15	LecB	89±54	330±49	0.728
E4-Gal	0.25	LecA	593±182	925±150	0.962
E4-Gal	0.50	LecA	369±50	766±46	0.989
E4-Gal	0.50	LecB	n.d.	n.d.	n.d.
E4-SA	0.50	LecA	n.d.	n.d.	n.d.

The data sets were analyzed and fitted to a Langmuir monosite isotherm model generating an equilibrium dissociation constant, K_d, maximum specific binding, F_{max} and a correlation coefficient R^2. N.d. weak binding with SDs exceeding the mean value. Results are averages with standard deviation from three independent repeats (n=3).

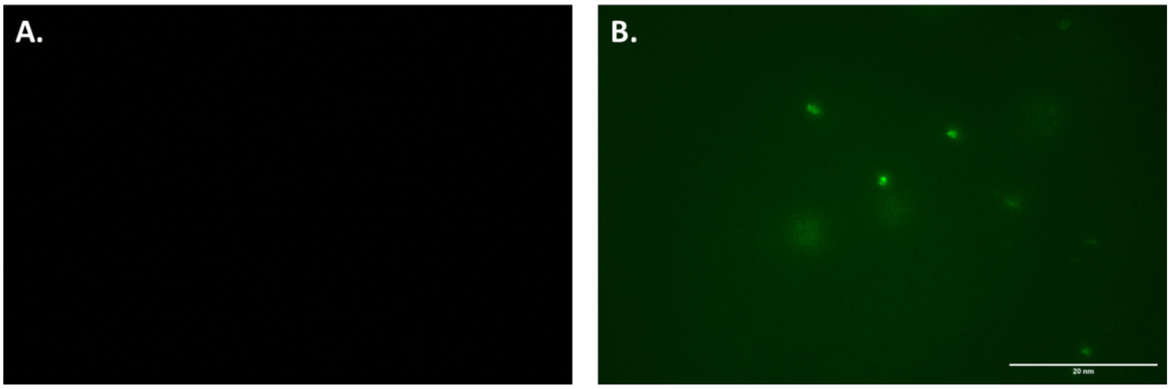

Figure S10. Fluorescence imaging using a WB filter on (A) $\chi_{E4\text{-}Gal}$ = 0.50 reference and (B) $\chi_{E4\text{-}Gal}$ = 0.50 incubated with LecA-FITC and rinsed with HEPES buffer at pH 8.

2.5 Evidence supporting the identity of new compounds

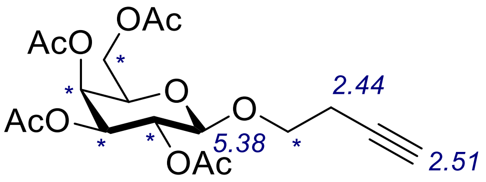

Figure S11. Assignment of 1H NMR chemical shift values (ppm) of 2. *Peaks at 5.10-3.67 ppm (8H total). #Peaks at 2.15-1.94 (12H total).

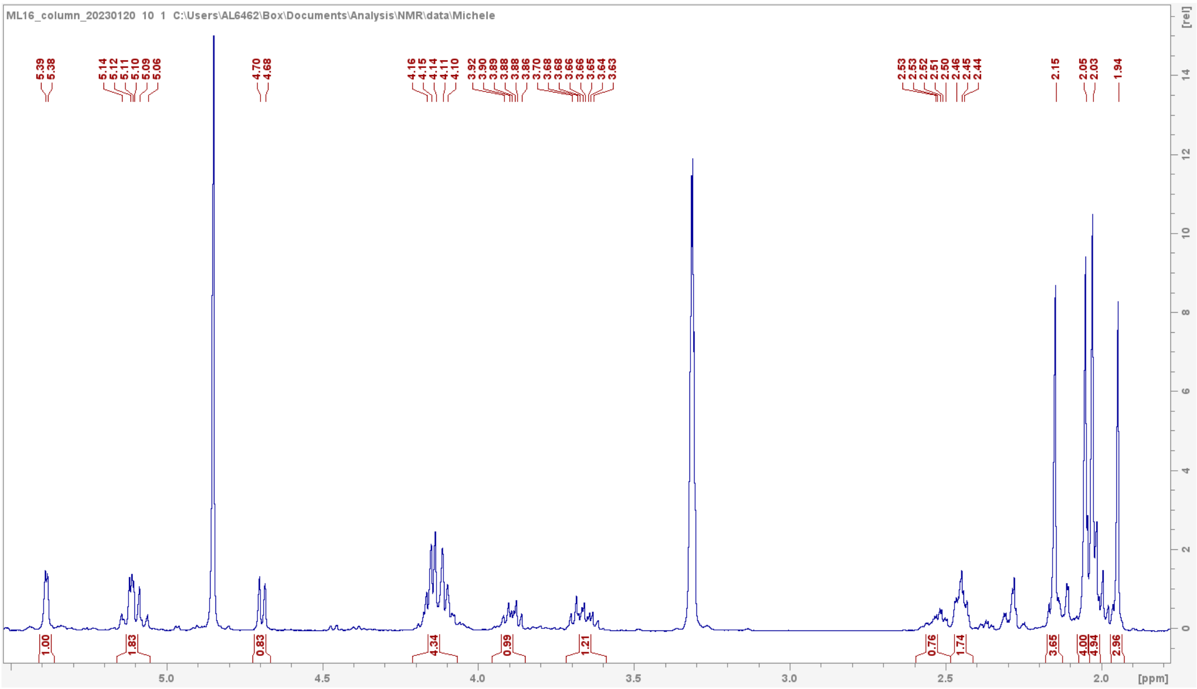

Figure S12. ^{1}H NMR spectrum of **2** (400 MHz, CD_3OD).

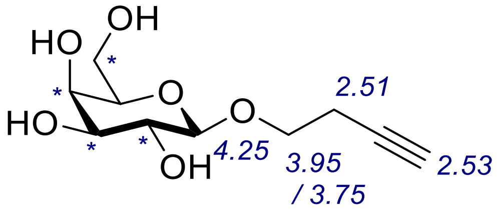

Figure S13. Assignment of ^{1}H NMR chemical shift values (ppm) of **3**. *Peaks at 3.67-5.10 ppm (6H total).

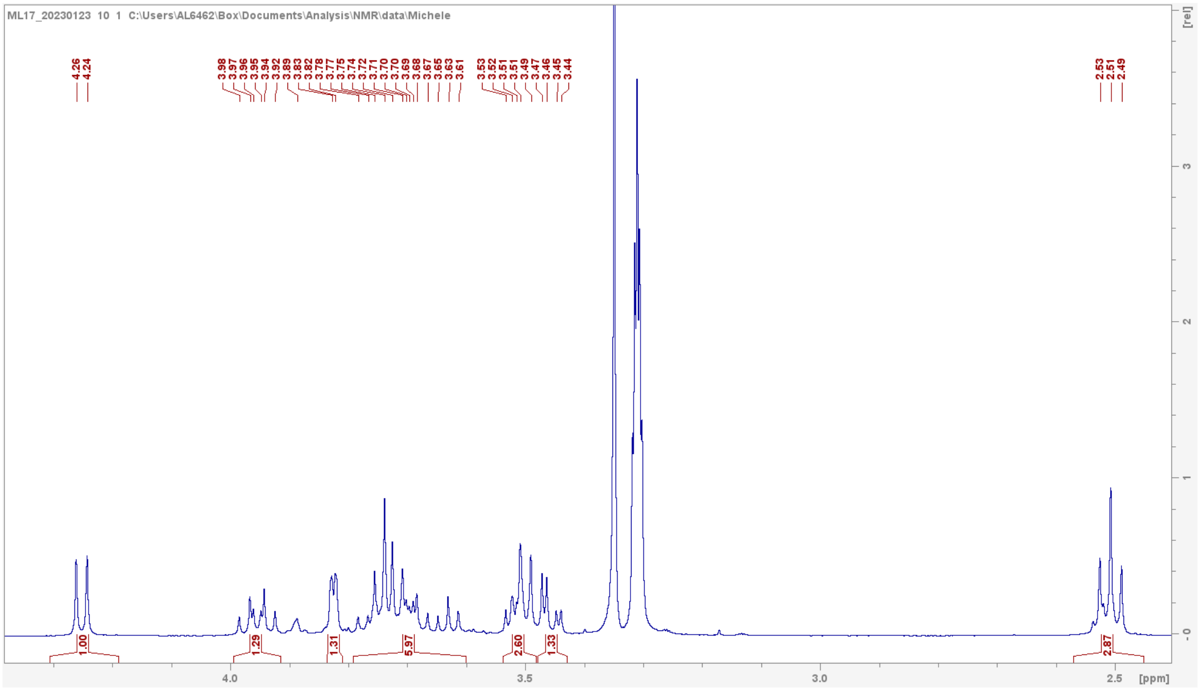

Figure S14. ^{1}H NMR spectrum of **3** (400 MHz, $CDCl_3$).

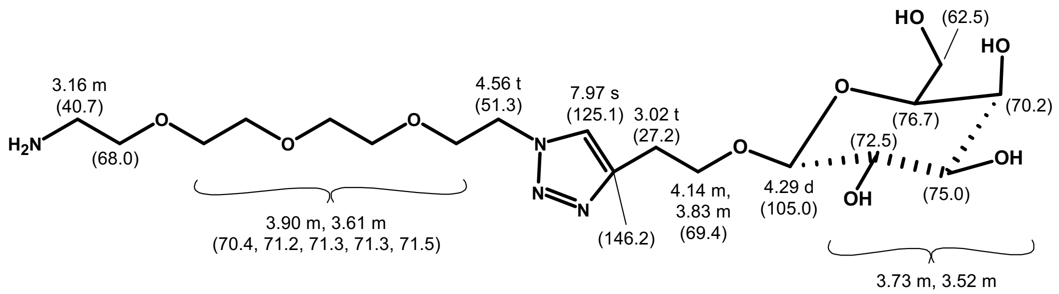

Figure S15. Assignment of ^{1}H and (^{13}C) chemical shift values (ppm) of **7**. The chemical shifts for the α-galactose anomeric carbon are commonly observed around 100.0 ppm, accompanied by a 4.80 ppm peak, corresponding to the anomeric proton. In the case of β-anomer, the corresponding signals are found at 105.0 (^{13}C) and 4.20 (^{1}H) ppm respectively. The stereochemistry of the galactose results further in a positive optical rotation for solutions of the α-anomer and negative values for β-galactose species.

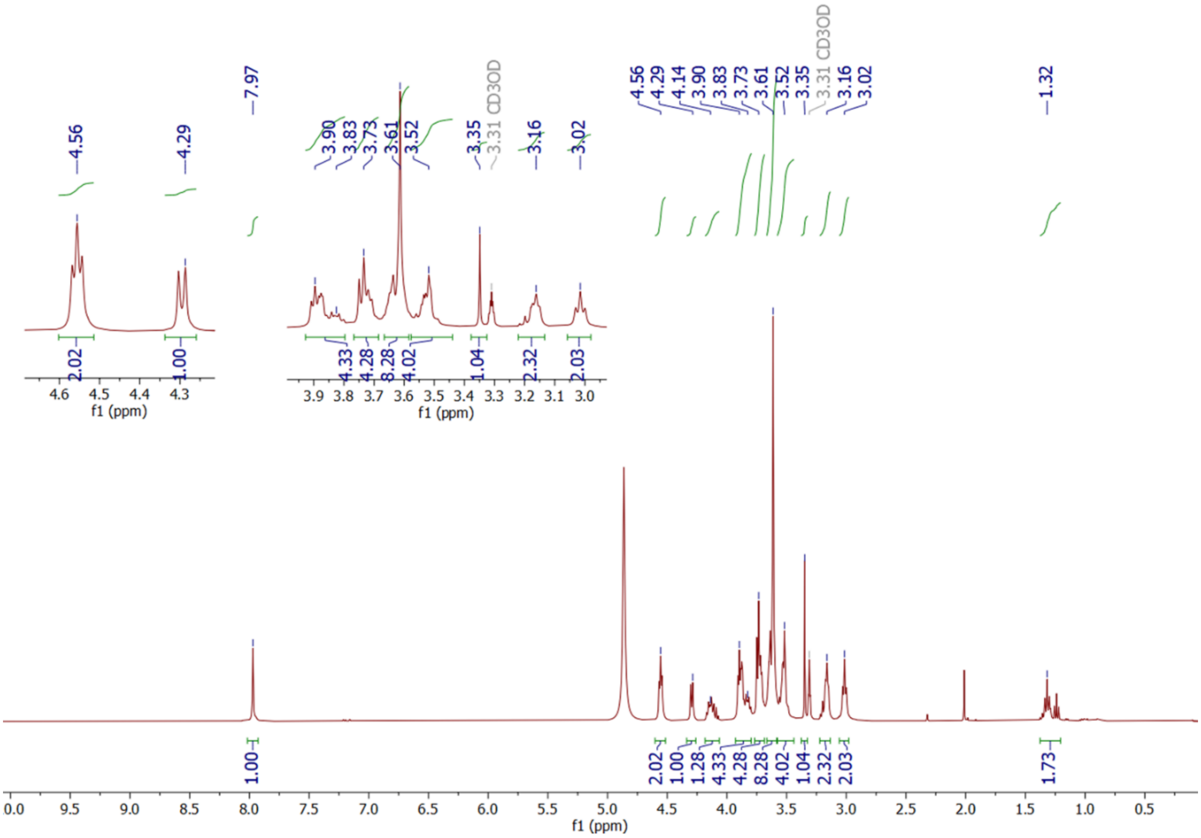

Figure S16. ^{1}H NMR spectrum of **7** (400 MHz, CD_3OD).

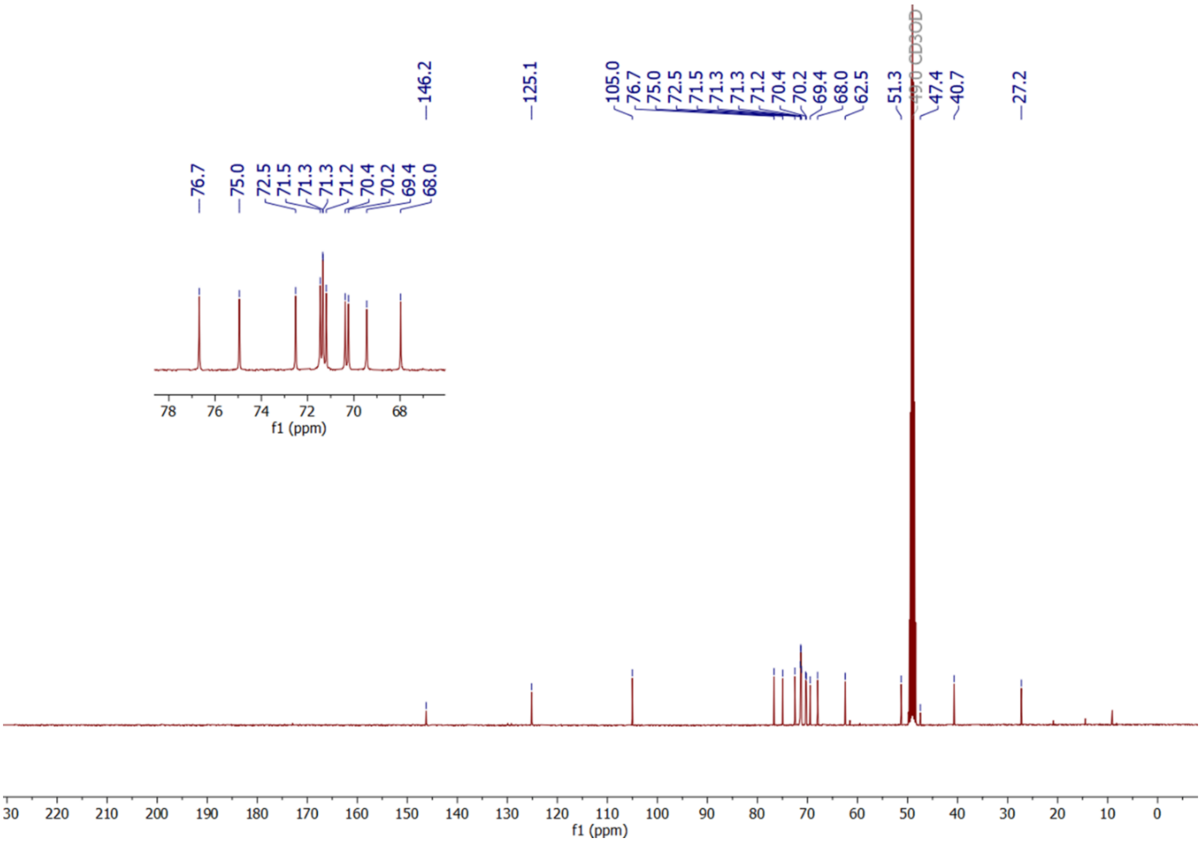

Figure S17. ^{13}C NMR spectrum recorded for **7** (400 MHz, CD_3OD).

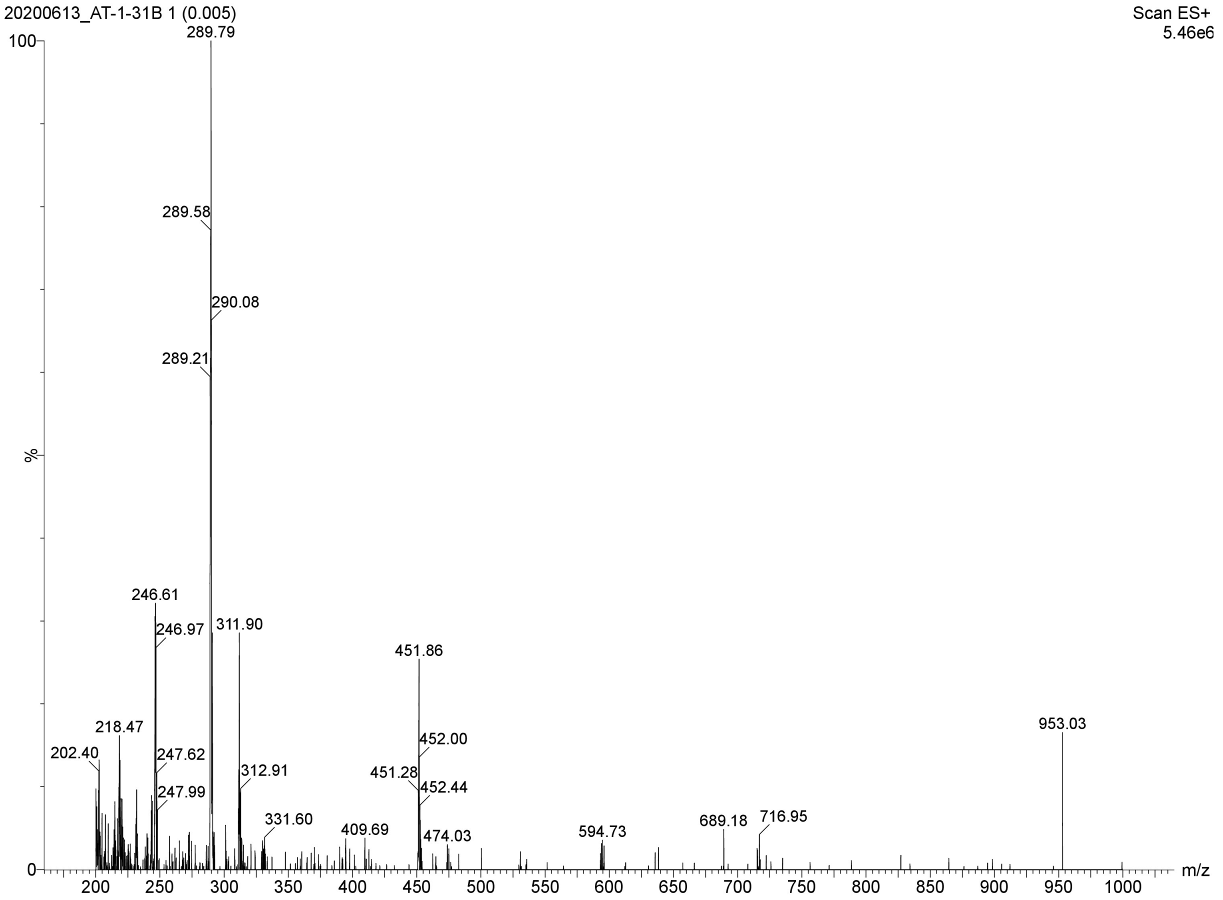

Figure S18. ESI mass spectrum of 7.

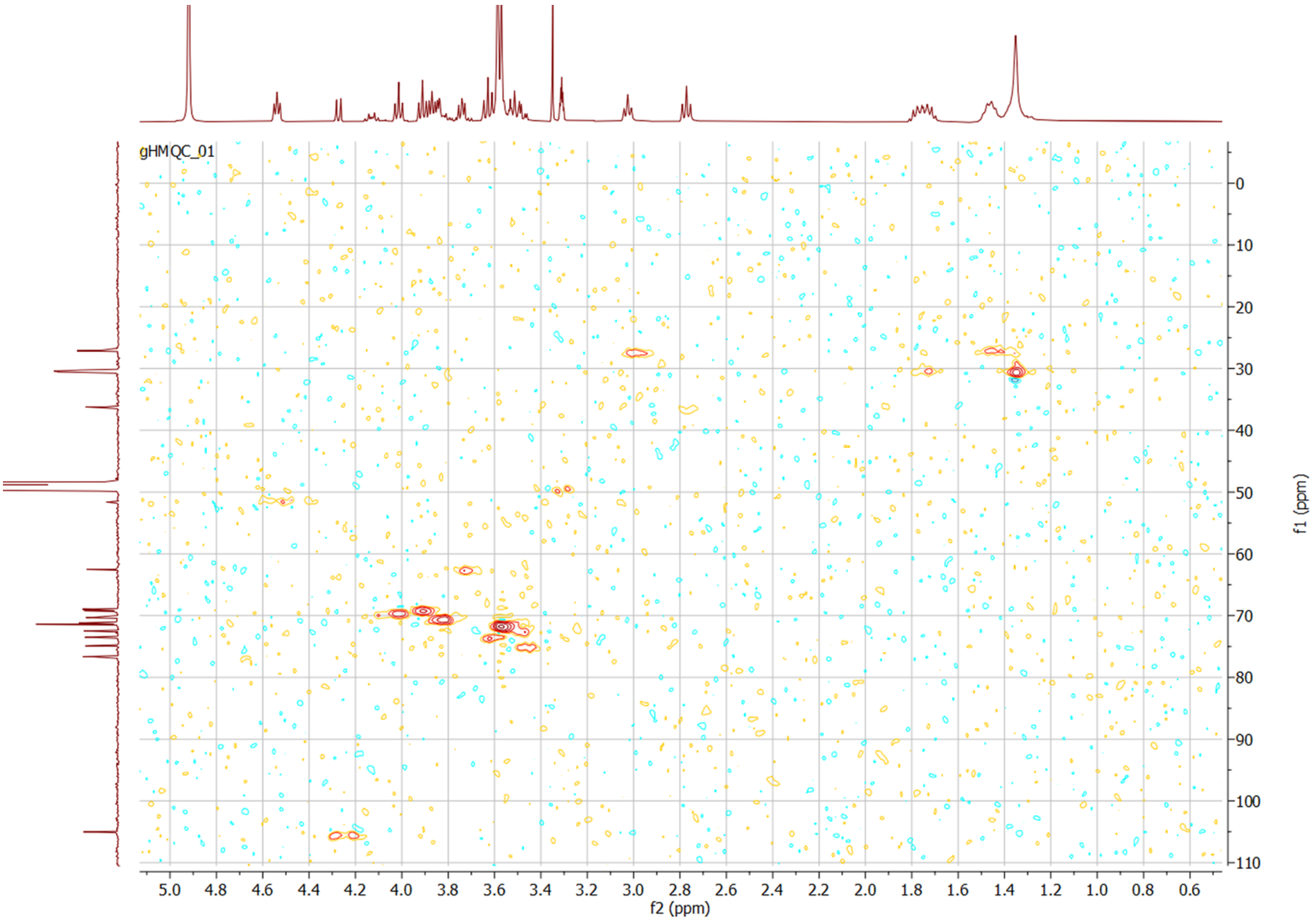

Fig. S19. ^{1}H-^{13}gHMQC spectrum recorded for **7**.

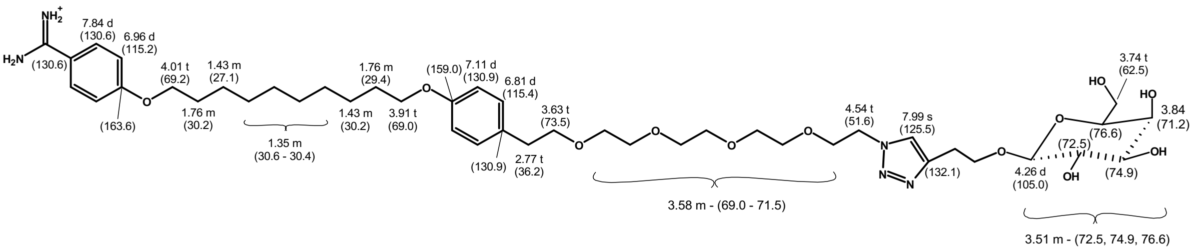

Figure S20. Assignment of ^{1}H and (^{13}C) chemical shift values (ppm) of **5**. The chemical shifts for the α-galactose anomeric carbon are commonly observed around 100.0 ppm, accompanied by a 4.80 ppm peak, corresponding to the anomeric proton. In the case of β-anomer, the corresponding signals are found at 105.0 (^{13}C) and 4.20 (^{1}H) ppm respectively. The stereochemistry of the galactose results further in a positive optical rotation for solutions of the α-anomer and negative values for β-galactose species.

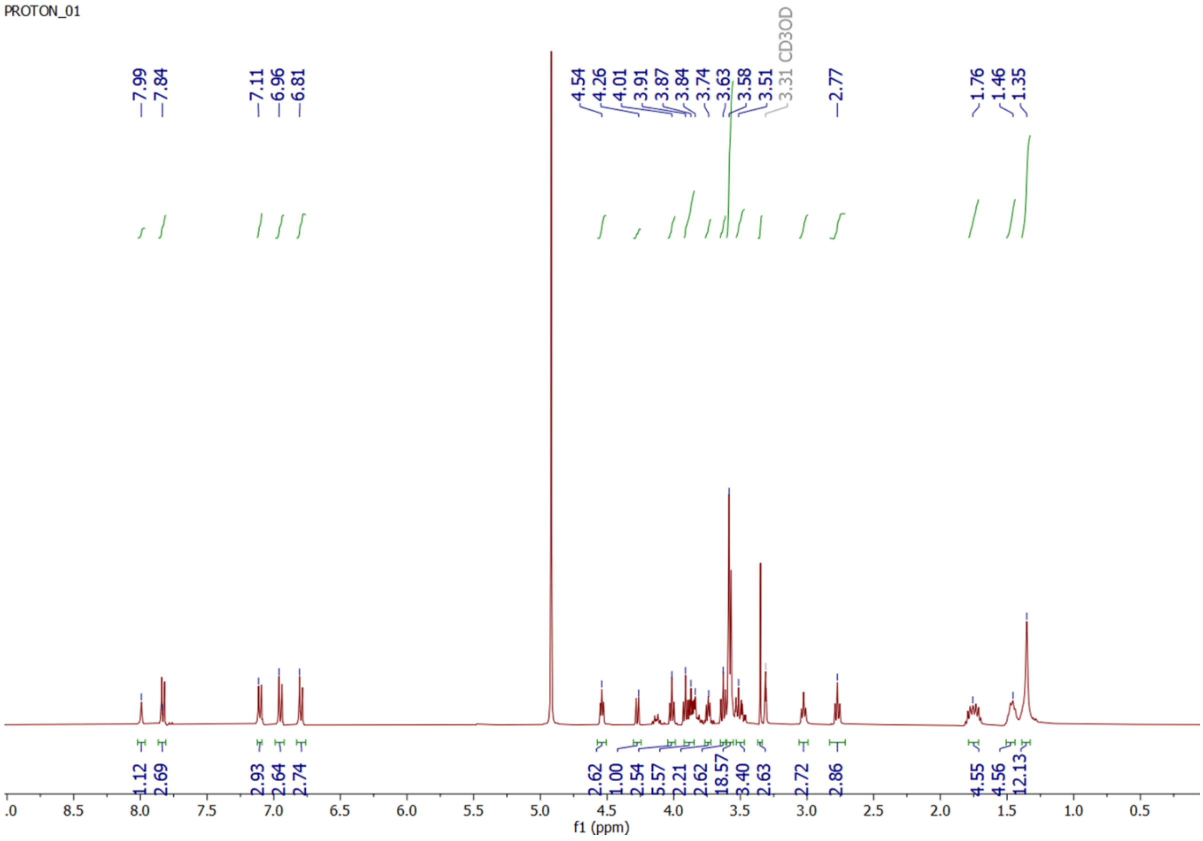

Figure S21. ^{1}H NMR spectrum of **5** (400 MHz, CD_3OD).

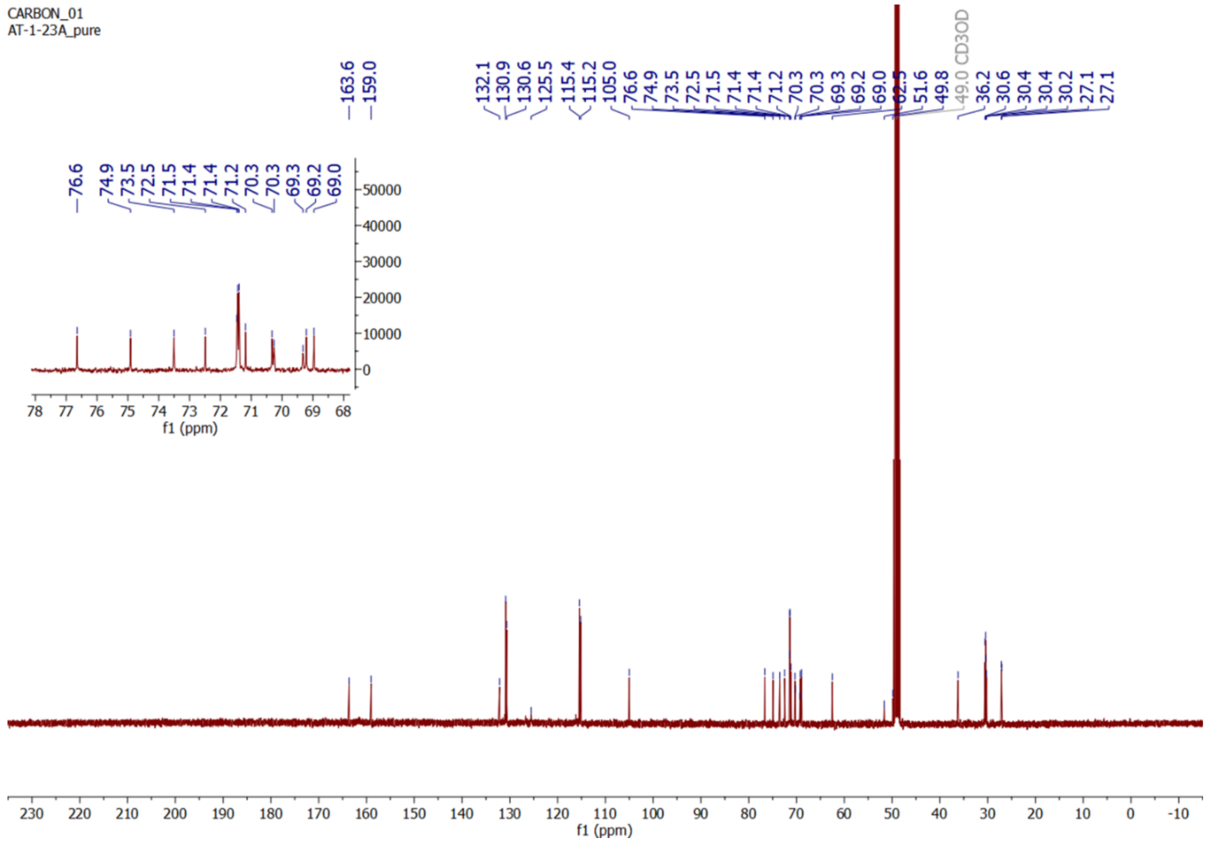

Figure S22. ^{13}C NMR spectrum recorded for 5 (400 MHz, CD_3OD).

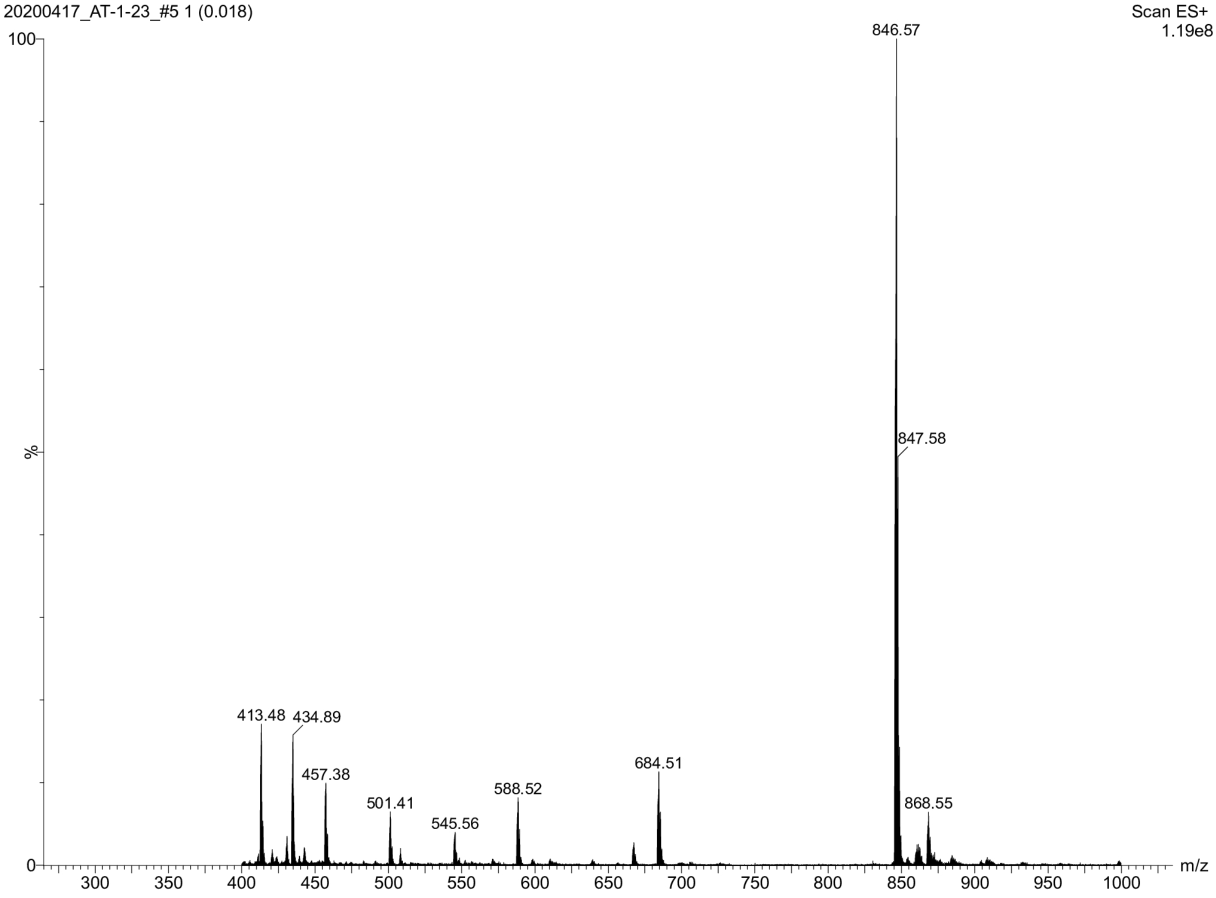

Figure S23. ESI mass spectrum of 5.

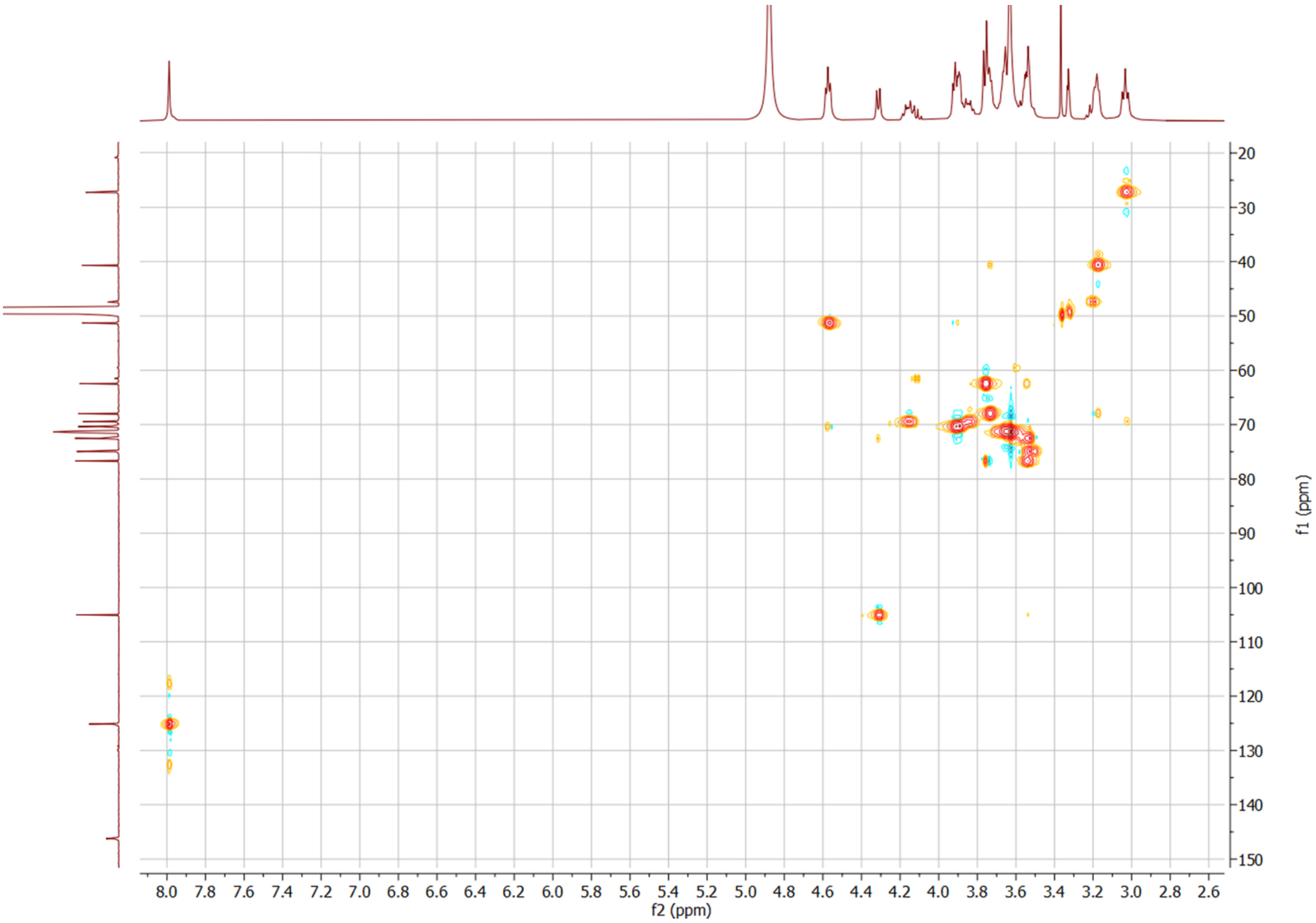

Fig. S24. ^{1}H-^{13}C HSQC spectrum recorded for **5**.
